# Machine Learning Quantitative Structure–Property Relationships as a Function of Ionic Liquid Cations for the Gas-Ionic Liquid Partition Coefficient of Hydrocarbons

**DOI:** 10.3390/ijms23147534

**Published:** 2022-07-07

**Authors:** Karl Marti Toots, Sulev Sild, Jaan Leis, William E. Acree, Uko Maran

**Affiliations:** 1 Department of Chemistry, University of Tartu, 14a Ravila Street, 50411 Tartu, Estonia; karl.marti.toots@ut.ee (K.M.T.); sulev.sild@ut.ee (S.S.); jaan.leis@ut.ee (J.L.); 2 Department of Chemistry, University of North Texas, 1155 Union Circle Drive #305070, Denton, TX 76203, USA; bill.acree@unt.edu

**Keywords:** Ionic liquid, QSPR, gas-ionic liquid partition coefficient, molecular interactions, support vector regression, gaussian process regression, multiple linear regression

## Abstract

Ionic liquids (ILs) are known for their unique characteristics as solvents and electrolytes. Therefore, new ILs are being developed and adapted as innovative chemical environments for different applications in which their properties need to be understood on a molecular level. Computational data-driven methods provide means for understanding of properties at molecular level, and quantitative structure–property relationships (QSPRs) provide the framework for this. This framework is commonly used to study the properties of molecules in ILs as an environment. The opposite situation where the property is considered as a function of the ionic liquid does not exist. The aim of the present study was to supplement this perspective with new knowledge and to develop QSPRs that would allow the understanding of molecular interactions in ionic liquids based on the structure of the cationic moiety. A wide range of applications in electrochemistry, separation and extraction chemistry depends on the partitioning of solutes between the ionic liquid and the surrounding environment that is characterized by the gas-ionic liquid partition coefficient. To model this property as a function of the structure of a cationic counterpart, a series of ionic liquids was selected with a common bis-(trifluoromethylsulfonyl)-imide anion, [Tf2N]^−^, for benzene, hexane and cyclohexane. MLR, SVR and GPR machine learning approaches were used to derive data-driven models and their performance was compared. The cross-validation coefficients of determination in the range 0.71–0.93 along with other performance statistics indicated a strong accuracy of models for all data series and machine learning methods. The analysis and interpretation of descriptors revealed that generally higher lipophilicity and dispersion interaction capability, and lower polarity in the cations induces a higher partition coefficient for benzene, hexane, cyclohexane and hydrocarbons in general. The applicability domain analysis of models concluded that there were no highly influential outliers and the models are applicable to a wide selection of cation families with variable size, polarity and aliphatic or aromatic nature.

## 1. Introduction

Ionic liquids (ILs) are a special class of chemical compounds that consist of ions and commonly refer to organic salts with a low melting point [1]. The combination of exclusive properties of ionic liquids, such as extremely low vapor pressure, high polarity and thermal stability, has been a major incentive behind their study as solvents and electrolytes in synthesis [2,3,4], catalysis [2,5,6], electrochemistry [7,8,9,10,11,12,13,14], extraction and separation chemistry [4,15,16,17,18,19,20,21] and in many other applications [22,23,24,25,26]. Applications of ionic liquids often involve complex molecular systems where IL is in contact with other organic compounds. For such systems, the gas-ionic liquid partition coefficient is an important measure that characterizes the distribution of an organic compound between an ionic liquid and the surrounding environment [27]. In order to optimize partition coefficients for specific applications, new ionic liquids are continuously produced [28,29,30,31,32,33]. Thereby, characterizing the influence of the ionic counterparts of the IL and constructing gas-ionic partition coefficient models based on the molecular structure of ion counterparts is essential in order to design application-targeted ionic liquids as rapidly, cost-effectively and as precisely as possible.

The gas-ionic liquid partition coefficient, *K*, quantifies the distribution of a chemical compound between a gas phase and an ionic liquid [34]:

(1) $$K=\frac{c_{IL}}{c_{G}},$$

where $c_{G}$ and $c_{IL}$ are compound concentrations in the gas phase and the ionic liquid, respectively. The partition coefficient is often provided in the logarithmic form, log *K*. The coefficient can be calculated from inverse gas-liquid chromatography (GLC) experiments as the ratio of the carrier gas volume used for solute elution to the stationary liquid phase volume. Experimental methods for finding *K* are laborious, costly, slow and require ample amounts of sufficiently pure compounds. Extensible screening efforts of compounds with application-suited log *K* are enabled using theoretical and computational approaches, such as quantitative structure–property relationships (QSPRs).

The literature shows that log *K* has been mostly modeled as a function of structure of organic compounds partitioning between gas and ionic liquid. Examples include the more commonly known Abraham solvation model [35,36,37,38,39,40,41,42,43,44,45] and a selection of linear and non-linear QSPR approaches [46,47]. Our recent research effort in this direction concentrated on modeling the gas-ionic liquid partition coefficient of a large variety of organic compounds in three ionic liquids [48]. We have previously developed a series of gas-liquid partition coefficient models for a general treatment of solubility in traditional organic solvents [49,50,51,52]. In another study, we modeled gas-liquid partition coefficients in methanol and ethanol [53]. The series of research has shown that QSPR approaches that employ theoretical molecular descriptors to study gas-liquid partition coefficients have been successful for many applications involved in physico-chemical [54,55], toxicological [56,57,58], biomedical [59,60] and material properties [61,62]. However, models for log *K* prediction in the scientific literature involving the ionic liquid’s molecular structure are incredibly sparse, with the only example being the ion-specific Abraham model [45]. Generally, QSPR-s concentrate on predicting the partitioning capacity of organic solvents within a specific ionic liquid [35,36,37,38,39,40,41,42,43,44,46,47]. These modeling efforts provide an understanding of partitioning interactions from the perspective of organic compounds, while the ionic liquid remains constant. On the other hand, it is equally important to understand the influence of ionic liquid structure on the partitioning properties. Thereby, characterizing the influence of the ionic counterparts of the IL and constructing gas-ionic partition coefficient models based on the molecular structure of ion counterparts are essential to design application-targeted ionic liquids as rapidly, cost-effectively and as precisely as possible. The present study tests the hypothesis that log *K* can also be modeled based on the partial structure of the ionic liquid. Advances in this computational modeling direction are beneficial to the ionic liquid research and development community, because such models improve the general understanding of ILs and help to design novel ILs while saving time and costs by reducing the need for experiments.

The study concentrates on modeling the gas-ionic liquid partition coefficients for three organic compounds hexane, cyclohexane and benzene in the series of ionic liquids with common bis(trifluoromethylsulfonyl)imide ([Tf2N]^−^) anion using linear and non-linear QSPR methods. [Tf2N]^+^ ILs are being extensively studied for various applications, for example as a media for supercapacitors [63], a heavy metal adsorbent [64], an anticancer agent [65] and gas solvent in sensors [66] among many other applications [67,68,69]. Variation in ionic counterpart makes it possible more specifically to understand how molecular interactions of partitioning by the ionic liquid and how to enable finding the application-appropriate ionic liquid. Hexane, cyclohexane and benzene are commonplace organic compounds, more specifically, hydrocarbons having the same number of carbon atoms but different molecular flexibility, saturation and a lack of electronegative atoms. Modeling the log *K* of these compounds in various ionic liquids with a constant anion allows characterizing the contributions of the structural properties of the IL cation with respect to three incrementally different solute environments. The interpretation of linear and non-linear QSPR models makes it possible to distinguish the main structural components of an ionic liquid and an organic solute that influence distribution between them.

## 2. Results

**Figure 1:**
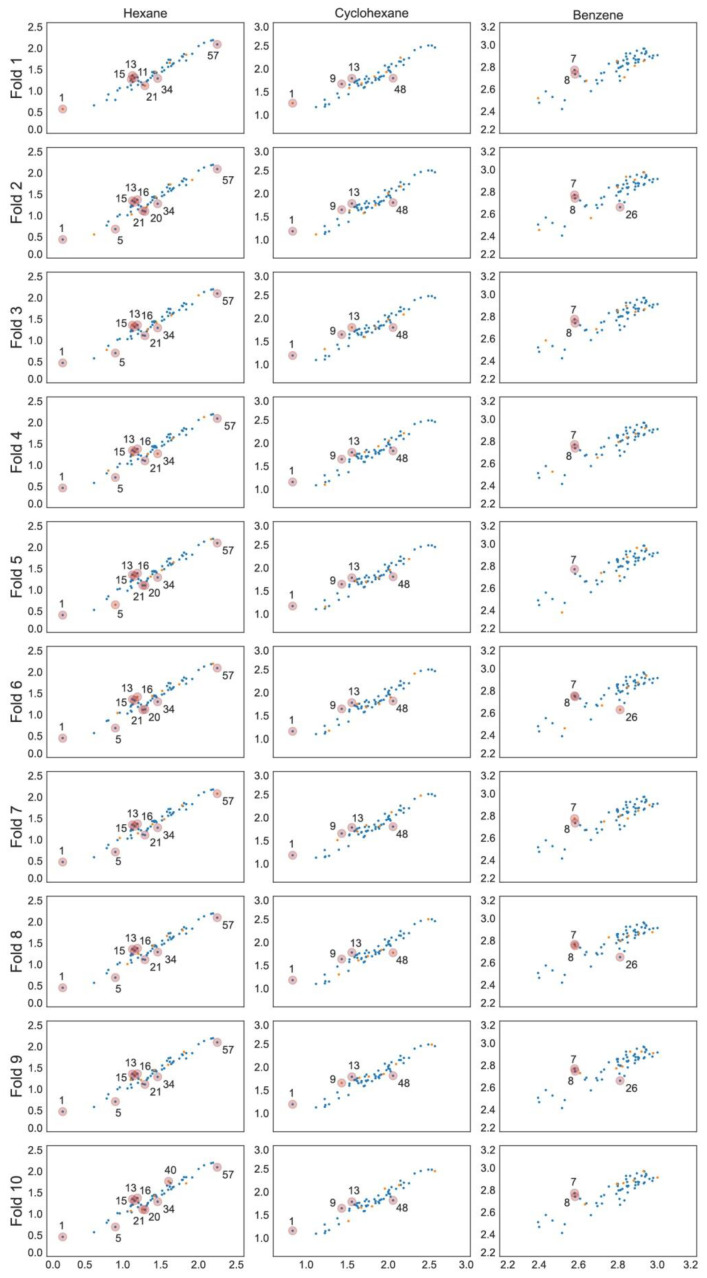
Predicted vs. experimental log *K* scatter plots for each MLR model with training set observations in blue and validation set values in orange. Compounds are numbered in ascending log *K* order (Tables S1–S3).

**Figure 2:**
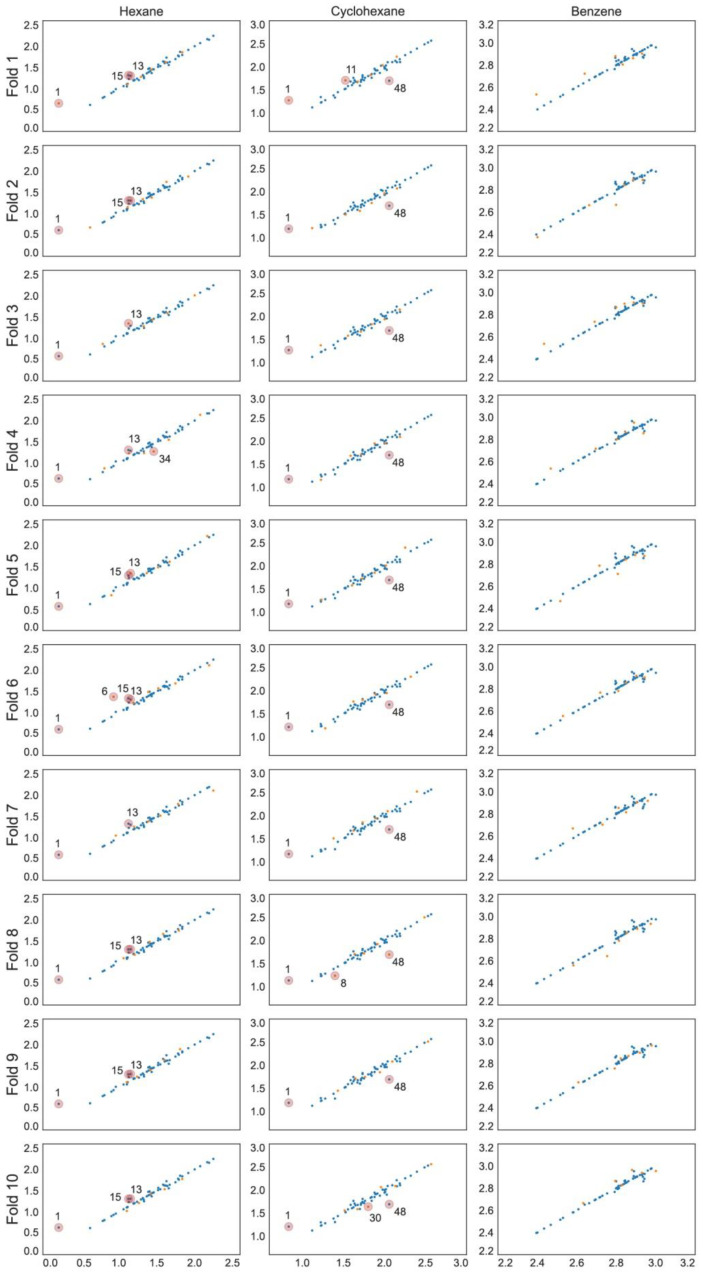
Predicted vs. experimental log *K* scatter plots for each SVR model with training set observations in blue and validation set values in orange. Compounds are numbered in ascending log *K* order (Tables S1–S3).

**Figure 3:**
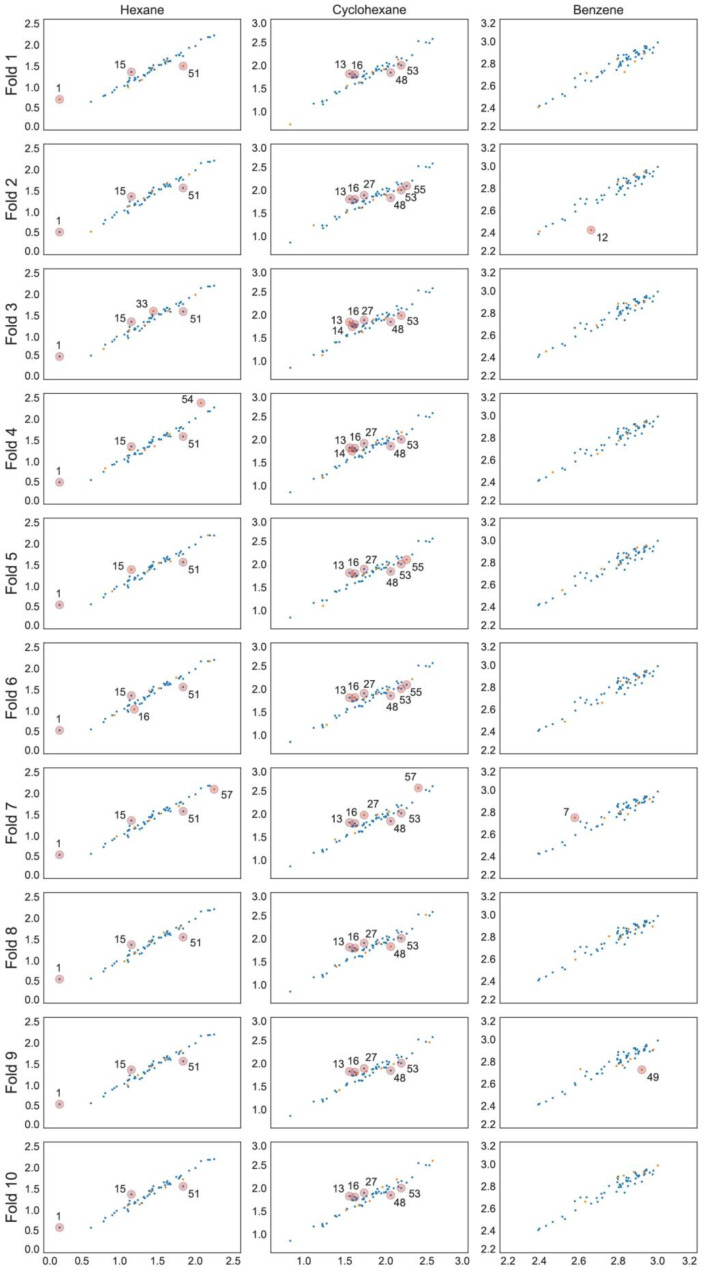
Predicted vs. experimental log *K* scatter plots for each GPR model with training set observations in blue and validation set values in orange. Compounds are numbered in ascending log *K* order (Tables S1–S3).

The optimal linear and non-linear (hyperparameters in Table 1) models found for hexane and cyclohexane data series showed cross-validated *r*^2^ values in the range 0.89 … 0.93. The linear and non-linear models for the benzene data series resulted in cross-validated *r*^2^ values in the range 0.72 … 0.85. The RMSE values for all models are within 0.05 to 0.11. All models include three to five parameters and the individual training and cross-validation statistics show high predictive capability on all validation folds (Table 2), which can also be seen from the experimental to predicted log *K* plots (Figure 1, Figure 2 and Figure 3). 

### 2.1. Models for Hexane in [Tf2N]^–^ Ionic Liquids

The final MLR model (Equation (2) of log *K* for hexane in [Tf2N]^−^ ionic liquids (MLRh) contained five molecular descriptors and had a cross-validation r^2^_cv10_ of 0.919. The selected descriptors included the following: *VE2_A* (average coefficient of the last eigenvector from the distance matrix), *AATS7s* (averaged Moreau–Broto autocorrelation of lag 7 weighted by intrinsic state), *ATSC0s* (centered Moreau–Broto autocorrelation of lag 0 weighted by intrinsic state), *AATSC2dv* (averaged and centered Moreau–Broto autocorrelation of lag 2 weighted by valence electrons) and *Xpc-4d* (4-ordered Chi path-cluster weighted by sigma electrons).

logK=1.417

−0.326 VE2_A

(2) −0.089 AATS7s

−0.126 ATSC0s 

+0.06 AATSC2dv

−0.133 Xpc-4d

The optimal five-parameter SVR model for hexane in [Tf2N]^–^ ionic liquids (SVRh) showed a cross-validation r^2^_cv10_ of 0.926, which is slightly higher than the statistics of the linear model. The selected molecular descriptors all were different in comparison with MLR model: *SMR_VSA5* (sum of Crippen–Wildman molar refractivity of atoms with van der Waals surface area 2.45–2.75), *AATSC0s* (averaged and centered Moreau–Broto autocorrelation of lag 0 weighted by intrinsic state), *SpMAD_D* (spectral mean absolute deviation from distance matrix), *AATS6m* (averaged Moreau–Broto autocorrelation of lag 6 weighted by mass) and *Xc-5d* (5-ordered Chi cluster weighted by sigma electrons).

In the GPR model for hexane in [Tf2N]^−^ ionic liquids (GPRh), the optimal model was found at four parameters with a cross-validation coefficient of determination r^2^_cv10_ of 0.924. Two of the molecular descriptors (*SMR_VSA5*, *Xpc-4d*) in the model were the same as in SVR and MLR models, respectively. The other two descriptors did not occur before: *GATS1s* (Geary coefficient of lag 1 weighted by intrinsic state) and *ATSC1are* (centered Moreau–Broto autocorrelation of lag 1 weighted by Allred–Rochow EN).

### 2.2. Models for Cyclohexane in [Tf2N]^–^ Ionic Liquids 

In the optimal MLR model (Equation (3)) for cyclohexane in [Tf2N]^–^ ionic liquids (MLRc), cross-validation r^2^_cv10_ was calculated to be 0.891. The model consisted of four descriptors: *VE2_A*, *GATS7Z* (Geary coefficient of lag 7 weighted by atomic number), *ATSC0s* and *Xpc-4d*.

logK=1.808

−0.329 VE2_A

(3) −0.101GATS7Z

−0.153 ATSC0s 

−0.126 Xpc-4d

The optimal SVR model derived for cyclohexane in [Tf2N]^−^ ionic liquids (SVRc) showed a cross-validation r^2^_cv10_ of 0.910. The four descriptors in the model were *SMR_VSA5*, *Xc-5d*, *AATSC0s* and *AATS7m* (averaged Moreau–Broto autocorrelation of lag 7 weighted by mass).

Using five descriptors, the GPR model for cyclohexane in [Tf2N]^–^ ionic liquids (GPRc) achieved a cross-validation r^2^_cv10_ of 0.903. The model consisted of molecular descriptors that were not present in previous models: *SLogP* (Wildman–Crippen LogP), *Xpc-4dv* (4-ordered Chi path-cluster weighted by valence electrons), *AATS0s* (averaged Moreau–Broto autocorrelation of lag 0 weighted by intrinsic state), *MATS8c* (Moran coefficient of lag 8 weighted by Gasteiger charge) and *AATSC6se* (averaged and centered Moreau–Broto autocorrelation of lag 6 weighted by Sanderson electronegativity).

### 2.3. Models for Benzene in [Tf2N]^–^ Ionic Liquids 

Regarding the MLR model for benzene in [Tf2N]^−^ ionic liquids (MLRb), the optimal model (Equation (4)) is characterized by a cross-validation r^2^_cv10_ of 0.717. Using the OMP algorithm, three descriptors were selected for the model: *AATS0s* (averaged Moreau–Broto autocorrelation of lag 0 weighted by intrinsic state), *GATS2dv* (Geary coefficient of lag 2 weighted by valence electrons) and *GATS3m* (Geary coefficient of lag 3 weighted by mass). None of them were present in the model for two other hydrocarbons.

logK=2.791

(4) −0.131 AATS0s 

−0.055 GATS2dv 

−0.042 GATS3m 

In the case of the SVR model for benzene in [Tf2N]^−^ ionic liquids (SVRb), however, the four-parameter model showcased a cross-validation r^2^_cv10_ of 0.851. The model included different set of the descriptors: Mi (mean of constitutional weighted by ionization potential), *ATSC1s* (centered Moreau–Broto autocorrelation of lag 1 weighted by intrinsic state), *GATS2pe* (Geary coefficient of lag 2 weighted by Pauling electronegativity) and *AATSC8i* (averaged and centered Moreau–Broto autocorrelation of lag 8 weighted by ionization potential).

The three-parameter GPR model for benzene in [Tf2N]^–^ ionic liquids (GPRb) had a cross-validation r^2^_cv10_ of 0.788 with the descriptors *AATSC0s*, *GATS3Z* (Geary coefficient of lag 3 weighted by atomic number) and *MDEC-12* (molecular distance edge between primary C and secondary C).

## 3. Discussion

### 3.1. Descriptors Interpretation

The interpretation of descriptors selected into the final models allows the analysis of the molecular structural factors that influence the gas-ionic liquid partition and interactions influenced by the cationic counterparts. The relative importance and influence of individual descriptors were analyzed using standardized regression coefficients in multiple linear regression models (Equations (2)–(4)), and the permutation importance was used for the descriptors chosen for the linear and non-linear models (Table 3). The variation in structure of cationic counterpart and the influence to the solvent properties of ILs can be understood in the context of major solute–solvent intermolecular interactions. The grouping of molecular descriptors according to the related solvent-solute interactions [4] (Table 4) allows generalizing which structural properties of the cation in the IL are relevant in the final models and can be optimized in looking for IL with a new constitution. This grouping considers the following major solute–solvent interaction mechanisms: dispersion forces related to molecule size, shape and polarizability; Coulomb and dipolar interactions related to cation counterpart charge distribution; and hydrogen bonding interaction related to the presence of functional groups capable of hydrogen bonding. The MLR model regression coefficient analysis allows a detailed look into the relationships between the log *K* and molecular descriptors, and linking this to the solvent–solute interaction provides means to gain knowledge on partition mechanism. Analogously, the SVR and GPR descriptor permutation importance analysis provides the opportunity to examine the associations between the selected descriptor, log *K* and major solvent–solute interaction.

### 3.2. Linear Models Descriptors

Linear models for hexane (MLRh) and cyclohexane (MLRc) are very similar. They have three common descriptors: *VE2_A*, *ATSC0s* and *Xpc-4d*. All these three descriptors have negative regression coefficients (Equations (2) and (4)) with similar values when compared between these models. The descriptor with the highest regression coefficient value is *VE2_A*, which is inversely proportional to the size of a molecule and related to dispersion forces (Figure S1). The *Xpc-4d* descriptor is influenced on the extent of branching in the cation, which is also related to its size and shape (Figure S3), and it has a negative regression coefficient, suggesting that higher log *K* value is associated with less branching. When comparing two molecules with identical atom counts, a higher extent of branching will decrease the surface area of the molecule and, therefore, will weaken intermolecular dispersion force interactions. The significance of dispersion force interactions in linear models is expected because both hexane and cyclohexane are non-polar molecules and, therefore, exhibit the hydrophobic effect towards polar groups in the cations of the ILs. That could explain the strong influence of the dispersion interaction and higher relative solubility of hexane and cyclohexane in the IL with lower *Xpc-4d* values. The importance of hydrophobic effects is further supported by the negative regression coefficient of the autocorrelation descriptor *ATSC0s*, which has the highest values for hydroxyl and cyano functionalized cations, followed by the cations with the ether group (Figure S2). Therefore, the *ATSC0s* descriptor values are higher for cations with polar groups and due to a negative regression coefficient, the model predicts lower log *K* values for hydrogen-bonding capable polar cations. 

The remaining descriptors with slightly lower regression coefficients (Equations (2) and (4)) in the hexane and cyclohexane models were *AATSC2dv*, *AATS7s* and *GATS7Z*. The *AATS7s* (Equation (2), Figure S5) and *GATS7Z* (Equation (4), Figure S6) descriptors are similar and their values are lowest for small cations, because the calculation of the distance matrix for a cation requires atom pairs with seven or more bonds apart to produce non-zero descriptor values. These descriptors characterize different structural aspects, where the *AATS7s* descriptor values are highest for aromatic and hydroxyl group-containing cations, while the GATS7Z descriptor values are highest for cations with long alkyl chains, ethers and aromatic cations. The *AATSC2dv* with a positive regression coefficient (Equation (2)) is also higher for aromatic cations (Figure S4). Therefore, the contribution of *AATS7s* and *AATSC2dv* descriptors is reduced for aromatic cations due to opposite signs in the MLRh model (Equation (2)). The higher values of the *AATS7s* for alcohol further support the prediction of lower values for cations with polar functional groups in the MLRh model.

In the linear model for benzene in [Tf2N]^−^ ILs (MLRb), the most influential descriptor based on the standardized regression coefficient was AATS0s. The descriptor is based on the average intrinsic electrotopological state [70], which increases due to O and N atoms, and from atoms with more attached hydrogens and greater bond order in the cation (Figure S7). It follows that the smaller cations with hydroxyl, cyano and ester functional groups and π-systems have the largest values. Due to its negative regression coefficient (Equation (4)), the model predicts lower log *K* values for cations with functional groups that exhibit hydrogen bonding and stronger dipolar interaction. This descriptor is also present in the previous two models with negative regression coefficients. The *GATS2dv* and *GATS3m* had also negative standardized coefficients in the MLRb model (Equation (4)). The *GATS2dv* descriptor values were lower for cations with aromatic π-systems and high bond order groups (Figure S8). This appears to reduce the contribution from the *AATS0s* descriptor value for such cations. Additionally, a trend for cations with a shorter alkyl chain and otherwise identical structure showed increases in the *GATS2dv* value (for example [HexylMPip]^+^ < [PentylMPip]^+^ < [ButylMPip]^+^). In that light, *GATS2dvs* in combination with the AATS0s descriptor have a compound effect for cations with π-systems. The descriptor also takes into account the dispersion force-related alkyl chain length, which results in a slightly higher predicted log *K* value for cations with longer alkyl chains. *GATS3m* characterized the same dispersion force-related alkyl chain length contribution effect as discussed for the *GATS2dv* descriptor. Furthermore, cations with the longest alkyl side chains typically had the smallest *GATS3m* values with a few exceptions, such as phosphoniums (Figure S9). Moreover, this further evidences the negative correlation of the *GATS3m* descriptor relative to the dispersion force strength.

### 3.3. SVR Models Descriptors

The final SVR models for hexane (SVRh) and cyclohexane (SVRc) in [Tf2N]^−^ ILs are similar in terms of the selected descriptors. The *SMR_VSA5*, *AATSC0s* and *Xc-5d* descriptors are common in both models. In addition, the *AATS6m* descriptor in SVRh and *AATS7m* in SVRc have almost identical calculation schemes. The *SMR_VSA5* (Figure S10) and *SpMAD_D* (Figure S15) descriptors selected for the SVRh model are both proportional to the cation’s size. In addition, *SMR_VSA5* is based on molar refractivity, which is directly related to polarizability. Consequently, the descriptors are related to dispersion force strength, which has a strong influence in the model. Both descriptors are among the top three descriptors with the highest permutation importance scores (Table 3). Similarly, the permutation importance of the *SMR_VSA5* descriptor in the SVRc model is the highest. In the SVRh and SVRc models, the *AATSC0s* descriptor is also among the top three influential descriptors. This descriptor is highly correlated with the *AATS0s* and *ATSC0s* descriptors selected in the linear models. Therefore, the *AATSC0s* (Figure S11) descriptor characterizes the hydrogen bonding capability and dipolar interaction strength of the cationic part in ILs. The *Xc-5d* descriptor in the SVRh and SVRc models has a value of 0 for most cations in the data sets, and it identifies molecules with two bonded high branching atoms (Figure S12). Therefore, it characterizes branching in the cation and could relate to dispersion forces. The *AATS6m* (Figure S13) in SVRh and *AATS7m* (Figure S14) in SVRc descriptors identify regions of the cation with higher average atomic mass among adjacent atoms, which is influenced by the presence of heteroatoms and the proportion of hydrogens in the cation, relating to hydrogen bonding and dipolar interaction forces. The proportion of hydrogens decreases with longer alkyl chains as also evidenced by the increasing descriptor values for [M3BAm]^+^ < [HexM3Am]^+^ < [M3OAm]^+^, meaning that the descriptor values also relate to dispersion interaction strength in some parts.

The SVRb model descriptor’s permutation importance decreased in the order of *Mi* > *ATSC1s* > *GATS2pe* > *AATSC8i* (Figures S16–S19). The Mi descriptor is the sum of atomic contributions of ionization potentials, divided by the ionization potential of carbon. The descriptor is normalized by the atom’s count in the cation. Its values have a low variance from 1.111 to 1.162 (Figure S16). The descriptor is also influenced by cation sizes, where the smallest cations have the largest descriptor value, and cations with shorter alkyl side chains have higher Mi values. Consequently, the descriptor could take into account both cation size and charge distribution, where the size is related to the dispersion interaction strength, and the presence of hetero atoms is related to dipolar interactions. The second largest importance was for the *ATSC1s* descriptor, which has a similar calculation scheme with the *ATSC0s* and *AATSC0s* descriptors. This descriptor identifies parts of the cation with hydrogen bonding capability and higher dipolar action strength (Figure S17). The lower importance descriptors *AATSC8i* and *GATS2pe* are based on atom ionization potential and the Pauling electronegativity, respectively. The *AATSC8i* descriptor (Figure S19) is calculated from atomic ionic potentials as Mi descriptor and could account for similar molecular interactions. The *GATS2pe* descriptor identifies parts of the molecule with high differences in Pauling electronegativity over a two-bond distance within the molecule. Smaller descriptor values are characteristic for aromatic heterocyclic cations, and larger values for ammoniums or aliphatic heterocyclic cations. The *GATS2pe* descriptor could, therefore, account for the effect of the cation family.

### 3.4. GPR Models Descriptors

The *SMR_VSA5* and *Xpc-4d* descriptors of the GPRh model, which were already discussed earlier, had the highest permutation importance in the model and are related the model prediction to dispersion interaction strength. *GATS1s* (Figure S20) separates different cation families and groups the aromatic cations close together and the aliphatic heterocyclic cations close together. This descriptor could account for interactions that differ between the cation families. The *ATSC1are* (Figure S22) is based on Allred–Rochow electronegativity between adjacent atoms [71,72]. Consequently, it could identify polar bonds in the cations and act as a measure of dipolar interaction strength. 

The highest permutation importance descriptor *SLogP* (Figure S22) in the GPRc model is a measure of the lipophilicity of the cations. Lipophilicity and hydrophobicity of the cation could play a considerable role in the solubility properties of the IL. According to the permutation importance value, the next most important descriptor was *Xpc-4dv* (Figure S23), which identifies path-clusters in the cations and relates to extent of branching similarly to *Xpc-4d*. Moreover, the descriptor similarly accounts for structural features related to the dispersion interaction. In addition, *Xpc-4dv* seems to separate different cation families and has lower values for aromatic heterocyclic cations. The *AATS0s* descriptor has the third highest importance and appeared in the MLRb model. Therefore, the descriptor could relate to hydrogen bonding capability and dipolar interaction strength in this model. The other descriptors *AATSC6se* and *MATS8c* (Figures S24 and S25) had relatively low permutation importance, about 1–2 factors of ten smaller than for *SLogP* and consequently the descriptors are less impactful. Only a few cations have non-zero and non-negligible *AATSC6se* values and the *MATS8c* values are in a small range from −0.52 to 0.152. *AATSC6se* considers the Sanders electronegativity of atoms and the *MATS8c* is based on Gasteiger charge. Both descriptors might account for dipolar interaction capability of the cation to some length.

In the GPRb model, the highest permutation importance was achieved by *AATSC0s*, which similarly in previous analysis could account for hydrogen bonding and dipolar interaction capability of the cation. *GATS3Z* (Figure S26) had a similar permutation importance to *AATSC0s* and is almost identical to the *GATS3m* descriptor, which appeared in the MLRb model. Based on the *GATS3m* analysis, *GATS3Z* could account for dispersion force strength. Lastly, *MDEC-12* (Figure S27) had a slightly lower permutation importance in the model. The calculation is based on distance between primary and secondary carbon pairs in the cation graph. Generally, more such pairs in the cation accumulate towards a bigger descriptor value. Cations with long alkyl chains have the largest values. Consequently, the descriptor relates to cation size and accounts for dispersion force strength in the model as well.

### 3.5. Comparison of Models for Different Solutes in [Tf2N]^–^ ILs

The interpretation of the descriptors selected into linear and non-linear models indicates some similarities and differences in the relative importance of the major solvent-solute interactions with respect to log *K*. All the models have descriptors that relate to dispersion forces, Coulomb and dipolar interactions, and hydrogen bonding capability (Table 4). Based on the linear models, the descriptors with highest standardized regression coefficient were related to dispersion forces in the case of hexane and cyclohexane. For the benzene linear model, the highest but negative regression coefficient was for *AATS0s*, for which its value was the largest for the smaller cations with hydroxyl, cyano and ester functional groups and π-systems. The relation to dipolar interactions and hydrogen bonding was more important in the case of benzene considering linear models. As for the log *K* value, based on descriptor interpretation, the log *K* prediction for all linear models was positively correlated with dispersion interaction capability and negatively correlated with dipolar interactions and hydrogen bonding means. A similar conclusion can be derived from the interpretation of permutation of descriptors of non-linear models, where the dispersion force related descriptors had the highest permutation importance for hexane and cyclohexane. Moreover, for benzene, the dipolar interaction counterpart descriptors had the highest permutation importance. Over all models, the more sizable, non-polar and lipophilic cations with longer alkyl chains and less aromaticity are predicted to have higher log *K*. Structurally, hexane and cyclohexane are more flexible molecules while benzene is more rigid. Consequently, hexane can acquire an optimal conformation in the IL environment, while cyclohexane is less flexible in that sense and benzene has even fewer degrees of freedom. The interactions related to lipophilicity are affected by packing density of interacting molecules. The descriptors selected for hexane and cyclohexane models had some emphasis on lipophilicity and the GPRc model even contained SLogP, which is a direct measure of lipophilicity. Regarding the higher relative importance of dipolar interaction capability of the cation in benzene models, a possible interpretation is that the benzene–cation interaction might be more sensitive to polar groups present in the cation than for hexane and cyclohexane. Since the solute is competing with anion–cation interactions in the solution, the anion–cation interaction, which changes between the cations, could instead be weaker to produce a higher solubility. Consequently, based on the descriptor interpretation, the larger and non-polar cations exhibit a weaker ion-cation interaction and by that induce the increase in the log *K* of the solute.

### 3.6. Analysis of Outliers

**Figure 4:**
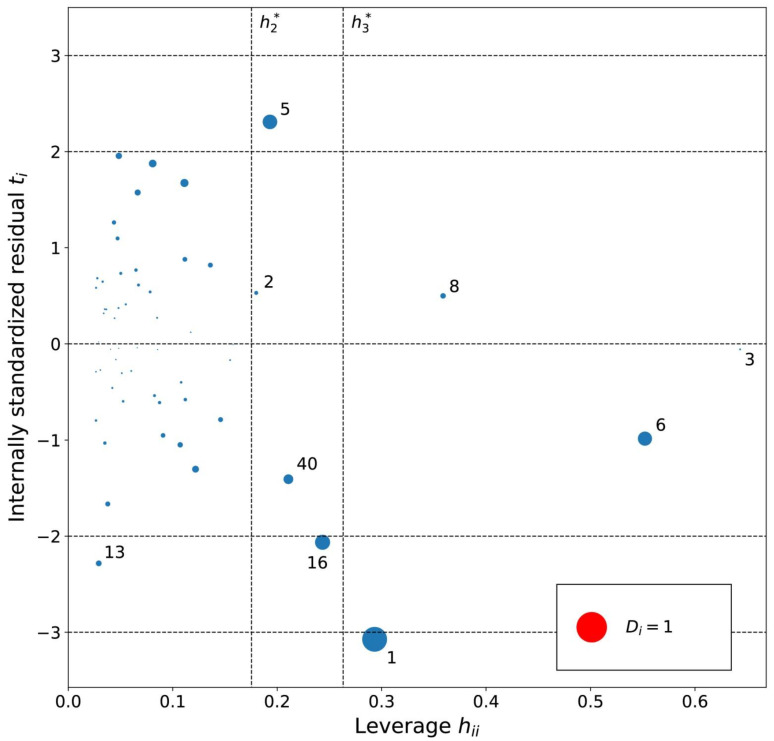
Influence plot for the hexane MLR model. The dotted horizontal lines distinguish possible outliers and vertical lines the high-leverage compounds. The point size is determined by the Cook’s distance (*D_i_*) value for the point. Cations are numbered in ascending log *K* order (Tables S1–S3).

**Figure 5:**
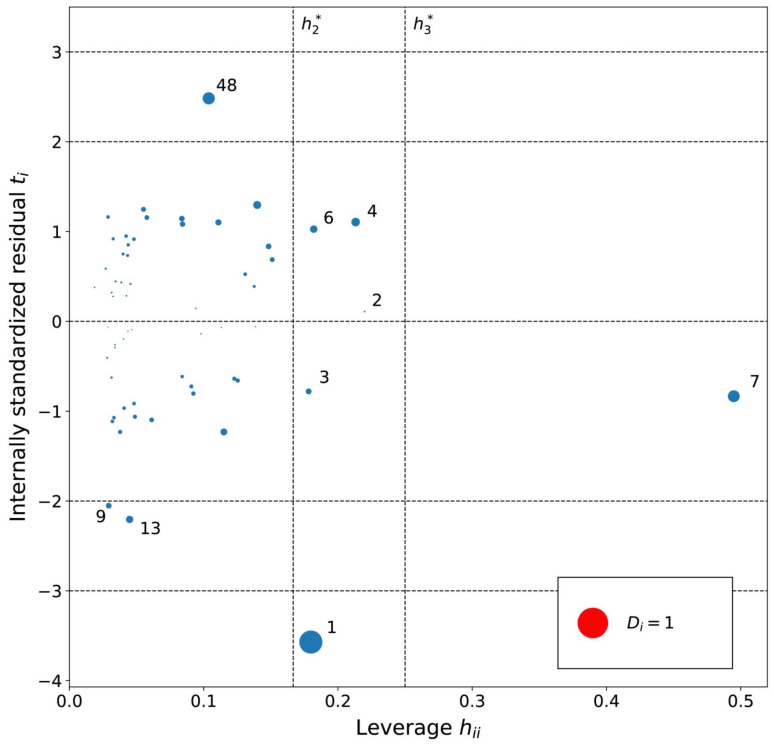
Influence plot for the cyclohexane MLR model. The dotted horizontal lines distinguish possible outliers and vertical lines the high-leverage compounds. The point size is determined by the Cook’s distance (*D_i_*) value for the point. Cations are numbered in ascending log *K* order (Tables S1–S3).

**Figure 6:**
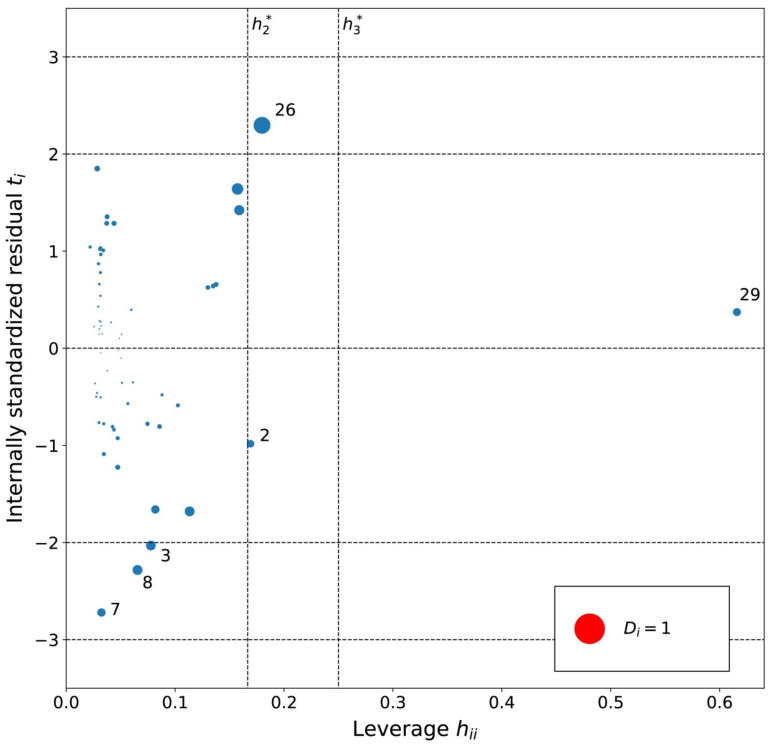
Influence plot for the benzene MLR model. The dotted horizontal lines distinguish possible outliers and vertical lines the high-leverage compounds. The point size is determined by the Cook’s distance (*D_i_*) value for the point. Cations are numbered in ascending log *K* order (Tables S1–S3).

The applicability domain of the MLR models was analyzed using an influence plot (Figure 4, Figure 5 and Figure 6) of standardized residuals against leverage values, where the size of points scales with Cook’s distance. On this plot horizontal and vertical lines identify thresholds for determining the moderate and high influence outliers. All Cook’s distance values were less than 1.0 indicating that the models do not contain highly influential outliers.

The number of data points with residuals of amplitude 2.0 or more standard deviations was roughly within the expected amount of 5% for the data set sizes. Normally distributed data with 60 samples are expected to contain about three such data points and residual analysis showed that all the linear models had four instances of residual amplitudes higher than 2.0.

Based on the model diagnostics and 10-fold cross-validation, these linear models are applicable for accurately predicting partition coefficients of hexane, cyclohexane, and benzene in the ionic liquids with the [Tf_2_N]^−^ anion. Overall, CCC (Table 2) evidenced that all the optimal models have potentially good predicting capabilities when applied to unforeseen data. 

The leverage values for the MLRh model (Figure 4) indicate four high leverage cations (Table 5): (1) [PrOHMMorp]^+^, (8) [4-CNBPy]^+^, (6) [C_1,9_(M_2_iPAm)_2_]^2+^ and (3) [EtOHM_3_Am]^+^. [PrOHMMorp]+ and [EtOHM_3_Am]^+^ are two out of the four alcohols in the data set and they also had relatively low log *K* values compared to the rest. In contrast to these similarities, the high leverage compounds were relatively unique to the data set. [PrOHMMorp]^+^ was one of the two morpholiniums and [C_1,9_(M_2_iPAm)_2_]^2+^ is also exclusive by being the only di-cation. Meanwhile, [4-CNBPy]^+^ is one out of the two cyano functionalized cations. Among the moderate leverage ILs were (2) [EtOHMIm]^+^, (5) [1-PrOHPy]^+^, (40) [TDC]^+^ and (16) [Et_3_S]^+^ (Table 5). The trend might imply that alcohols in the hexane series have some leverage on the model. However, two out of the four alcohols had low internally standardized residuals. Both [Et_3_S]^+^ and [TDC]^+^ have rare structural properties in the data set, where [Et_3_S]^+^ is the only sulfonium and [TDC]^+^ is the only cycloalkanylium and the only carbon cation, which could explain their moderate influence on the model.

The residual analysis determined four moderate or high residual cations for the MLRh model. The moderate residual cations were (16) [Et_3_S]^+^, (13) [BzMPyrr]^+^ and (5) [1-PrOHPy]^+^, and the (1) [PrOHMMorp]^+^ cation had a high negative internally standardized residual. The moderate residual [Et_3_S]^+^ cation was already indicated as a unique sulfonium in the leverage diagnosis, and it is also one of the smallest cations in the entire data series. The cations indicated by the residual analysis of the influence plot do not share an overarching common structural theme. The Cook’s distance values of the potentially influential data points were all below 1.0, where the [PrOHMMorp]^+^ cation had the highest Cook’s distance of 0.65. The [PrOHMMorp]^+^ log *K* value for hexane was the lowest out of all experimental log *K* values in the study, where the next lowest was [EtOHMIM]^+^ cations that were three-times higher experimental result. This makes the [PrOHMMorp]^+^ cation a significant outlier in terms of the provided experimental values and might explain why it has the most influence on the hexane MLR model. 

The cations with a moderate leverage in the MLRc model (Figure 5) were (1) [PrOHMMorp]^+^, (2) [CNMeM_2_iPAm]^+^, (3) [(Meo)_2_Im]^+^, (4) [EtOHM_2_iPAm]^+^, (6) [EtOHM_3_Am]^+^ and the (7) [C_1,9_(M_2_iPAm)_2_]^2+^ had highest leverage. Similarly to the hexane series, a common structural property of the significant leverage compounds is the presence of the hydroxyl group. Since the MLRh and MLRc models contained descriptors that account for similar structural properties, a similar pattern could be expected. [EtOHM_2_iPAm]^+^ and [EtOHM_3_Am]^+^ have a similar structure while being relatively small cations compared to the rest. In regards to the high leverage [C_1,9_(M_2_iPAm)_2_]^+^, the only di-cation can be expected to have unique interaction relative to cyclohexane molecules and the [Tf_2_N]^-^ counteranion that the linear model might not encapsulate so well.

Based on the residual analysis, the moderate residual cations were (9) [MeoeMMorp]^+^, (13) [BzPy]^+^ and (48) [M_3_BAm]^+^. Similarly to the hexane model, the (1) [PrOHMMorp]^+^ cation showed a high residual. Evidently, both of the morpholiniums appeared among the significant residual cations. The [PrOHMMorp]^+^ log *K* value for cyclohexane was again the lowest out of all experimental log *K* values in the cyclohexane data series. This might indicate that some structural features of the morpholiniums or interaction with its environment are more difficult to capture with the descriptors selected in the linear model. Other high residual cations for the MLRc model do not resemble a common structural theme.

From the leverage analysis of the influence plot for MLRb model (Figure 6), the following significant leverage cations were found: (2) [EtOHMIm]^+^, (26) [4-CNBPy]^+^, and (29) [Et_3_S]^+^. [Et_3_S]^+^ indicated the highest leverage and previous analysis already turned its attention to its rare structure with a sulfonium cation and small size, which could explain its possible influence here as well. The moderate leverage cations could have leverage due to hydroxyl ([EtOHMIm]^+^) and cyano ([4-CNBPy]^+^) functionalizations. No other common structural features are obvious and the Cook’s distances show that the significant leverage cations for the MLRb model are not highly influential.

An analysis of the internally standardized residuals for the MLRb model indicated no high residuals and four moderate residual cations: (3) [PrOHMMorp]^+^, (7) [BzPy]^+^, (8) [C_1,9_(M_2_iPAm)_2_]^2+^ and (26) [4-CNBPy]^+^. In the benzene data series, [PrOHMMorp]^+^ had the third-lowest log *K* value, which could explain its moderate residual. Secondly, the moderate residual of [C_1,9_(M_2_iPAm)_2_]^2+^ could be due to its unique di-cation nature. Furthermore, [4-CNBPy]^+^ is one out of the only two cyano functionalized cations used for the MLRb model, which might be the reason for its moderate residual. The residual analysis did not indicate a distinct pattern in the possible outliers for the benzene series and the Cook’s distances were all below 1.0, which demonstrates no highly influential outliers.

## 4. Materials and Methods

### 4.1. Data Set

The data set comprised three series of experimental gas-ionic liquid partition coefficients (log *K*) measured at 298 K for benzene, hexane and cyclohexane in various ionic liquids where bis(trifluoromethylsulfonyl)imide ([Tf2N]^–^) was the common anion (Tables S1–S3, Supplementary Material) [42,73,74,75,76,77,78,79,80,81,82,83,84,85,86,87,88,89,90,91,92,93,94,95,96,97,98,99,100,101,102,103,104,105,106,107,108,109]. These three data series had similar sizes with 57, 60 and 60 partition coefficients for hexane, cyclohexane and benzene, respectively. In addition, all three data series consisted of the same cations, except the hexane data set that did not have log *K* values for three cations. The cations of ionic liquids studied were diverse in terms of their molecular structure, including different cation families, functional groups, aliphatic or aromatic rings and aliphatic chain branching and length. Nearly half the cations were either the imidazolium or ammonium cation families. More sparsely represented were the pyrrolidiniums with 8, pyridiniums with 6 and piperidiniums with 5 cations, followed by other cation families with up to two representatives. Notable further functionalized cations were the 6 ethers, 5 alcohols and the 2 nitriles. Experimental gas-ionic liquid partition coefficient values ranged from 0.209 to 2.248 for hexane, 0.836 to 2.569 for cyclohexane and 2.395 to 3.001 for benzene.

### 4.2. Cation Structure and Data Series Preparation Workflow

**Figure 7:**
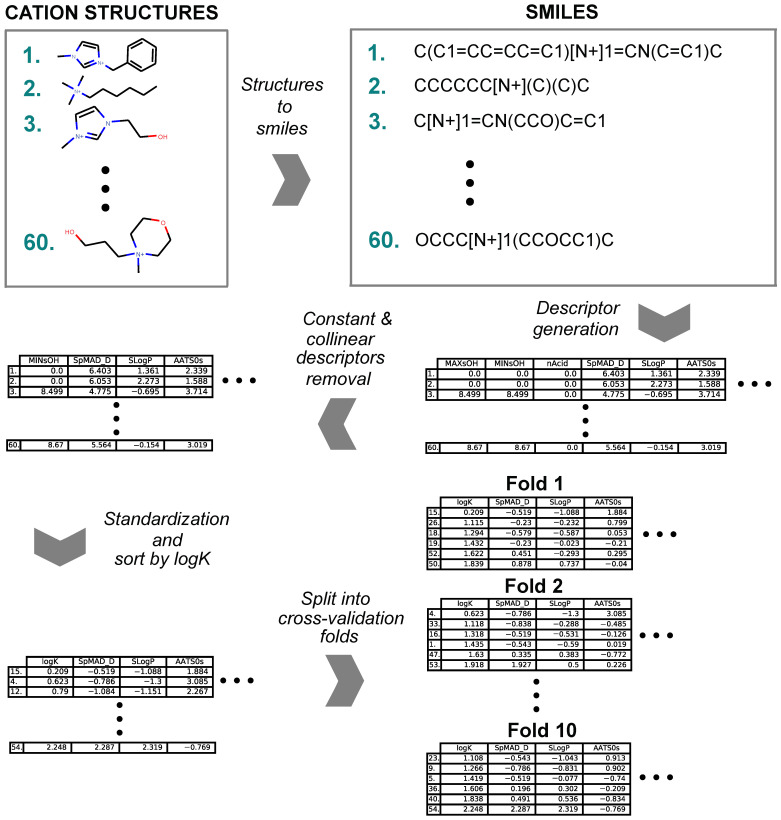
Data series preparation workflow.

The preparation of each data series for modeling followed a general workflow (summarized in Figure 7) that consisted of SMILES [110] creation for the cation part of the ionic liquid, descriptor generation, removal of redundant descriptors, standardization of descriptor values and subdividing for cross-validation. The names of the cations were used to create the corresponding SMILES representations. The molecular descriptors were calculated with the Mordred [71] library (version 1.1.1) that uses the rdkit [111] library (version 2018.09.3). The calculation resulted in 1613 2D molecular descriptors for each cation, which formed a cation-descriptor matrix. The redundant constant value descriptors and all but one in sets of collinear descriptors were removed from the solutes cation-descriptor matrix. The remaining descriptors were standardized to a mean of 0 and a standard deviation of 1. In the resulting matrices, the cations were sorted in the ascending order by log *K* values. Then, the cations were subdivided into ten folds where each fold consisted of every tenth cation in the log *K* sorted matrix. The described partitioning scheme resulted in ten data sets that contained evenly distributed partition coefficient values for 10-fold cross-validation. As a result of the preparation, three cation-descriptor matrices for benzene, hexane and cyclohexane all consisted of 1180 descriptors and 10-folds. 

### 4.3. Multiple Linear Regression

Multiple linear regression (MLR) is given by the linear combination of molecular descriptors with a modeled property $\hat{y}$ [112]:

(5) $$\hat{y}=\beta_{0}+\beta_{1}X_{1}+\beta_{2}X_{2}+\ldots +\beta_{k}X_{k},$$

where *β*_0_, *β*_1_, …, *β_k_* are regression coefficients and *X*_1_, *X*_2_, …, *X_k_* are molecular descriptors. The regression coefficients vector $\hat{\beta }$ is regularly calculated using the ordinary least squares method to minimize squared error [112]:

(6) $$\hat{\beta }={\left(X^{T}X\right)}^{-1}X^{T}y,$$

given experimental property values *y* and the molecular descriptor values of matrix *X*.

The orthogonal matching pursuit (OMP) algorithm is a bottom-up feature selection algorithm that selects a feature into a linear model on each iteration based on the correlation of the features to the residual of the linear model estimations [48,113]. OMP was used for the selection of features (molecular descriptors) into MLR models. The OMP algorithm is computationally efficient for the selection of descriptors that have a low correlation between each other and therefore accounts for potentially more varied chemical information with each selected descriptor. From the Scikit-learn [114] (version 0.24.2) library, the OrthogonalMatchingPursuit class was used as the implementation of OMP.

The expansion of the search space of the OMP algorithm and improvements in the selected combination of descriptors was achieved by iteratively expelling the highest correlated descriptor to log *K*. In order to procure a wider selection of linear models using OMP, after the initial selection, the descriptor with the highest correlation to log *K* was expelled and OMP was applied again. This process was repeated until the highest correlation to log *K* complied with R < 0.4. Out of the models with the same number of descriptors found by the OMP algorithm, the one with the highest coefficient of determination was deemed optimal. The number of descriptors selected started with one and the amount was incremented until the model did not show significant improvement. In this case, significant improvement was confirmed if the parameter with an extra descriptor corrected at least 20% of the prediction error according to the coefficient of determination.

### 4.4. Support Vector Regression

The support vector regression (SVR) algorithm solves a constrained optimization problem to find the optimal set of training points *x_sv_* called the support vectors, the regression coefficients, and the intercept in the SVR model [115]. The support vectors are equidistant from the computed regression line and mark the margin boundary termed the ε-tube around the regression line, where points outside the ε-tube are penalized in the optimization expression. 

The resulting model can account for non-linearity with respect to the features due to transformations via the kernel function [116]. The kernel function maps training points into higher dimensional space, which characterizes the similarity between two data points and can be chosen according to the modeling problem. The linear, polynomial, radial basis function (RBF) and sigmoid kernel are some of the commonly applied kernel functions. The SVR algorithm involves hyperparameters C and ε, which influence the optimization expression. The error penalization term C is generally chosen to be 1 but its value should be configured with attention to the input data, as it influences how much the errors σ_i_ penalize the optimization expression and in turn deviate the regression line. In addition to C and ε, the chosen kernel function may contain additional hyperparameters; for example, the RBF function can be tuned by changing a scale coefficient γ. 

The Scikit-learn [114] (version 0.24.2) library SVR class and the RBF kernel were used as SVR implementation. The prediction of the SVR model for a molecule with descriptors x is as follows:

(7) $$\hat{y}=\sum_{i\in SV}\alpha_{i}\;K\left(x_{i},x\right)+b,$$

where α are the regression coefficients of the support vector training points, $x_{i}$ includes descriptor values for the *i*-th support vector, *K* is the kernel function, and *b* is the intercept.

Descriptor selection for the SVR models followed a bottom-up scheme, where descriptors were selected into the model based on the highest 10-fold cross-validated validation coefficient of determination. After a new descriptor was selected into the model, all descriptors were also substituted one by one until the model did not improve anymore from any single parameter substitution. The concluded optimal model had its hyperparameters C, ε and γ tuned by refitting with a combination of hyperparameters from a grid of predetermined values (Table 6). Whether a higher parameter model was considered better was based on improvement in comparison to the optimal model with one less parameter. If the higher parameter model corrected at least 20% of the prediction error, more parameters were added until the model did not improve by 20% anymore.

### 4.5. Gaussian Process Regression

In the Gaussian process regression (GPR) method, a distribution over functions is defined using the training data, a covariance function and corresponding log *K* values [117]. The predictions from the fitted GPR model form a full predictive distribution with a mean and standard deviation at every point of the input space [117]. The GPR model predicted distribution for validation data points $x_{*i}$ can be calculated by evaluating the mean ${\bar{f}}_{*}$ and the covariance matrix $V\left[f_{*}\right]$ from the following [117]:

(8) $${\bar{f}}_{*}=K\left(X_{*},X\right){\left[K\left(X,X\right)\right]}^{-1}y,$$

(9) $$V\left[f_{*}\right]=K\left(X_{*},X_{*}\right)-K\left(X_{*},X\right){\left[K\left(X,X\right)\right]}^{-1}K\left(X,X_{*}\right),$$

where X is the training cation-descriptor matrix, $X_{*}$ is the validation cation-descriptor matrix, y is the vector of log *K* values corresponding to the training data points, and K(A, B) is the kernel dot product, e.g., the covariance function between the input matrices A and B [117]. The commonly used RBF kernel function maps the descriptors into an infinite-dimensional descriptor space; however, by only using dot products between kernel mapped inputs, the individual kernel-mapped descriptors do not need to be calculated [117].

Kernels used in constructing the GPR model have an effect on the derived space of functions [117]. Commonly applied kernels include the constant, white noise, dot product, polynomial, RBF kernel and combinations [117]. In this study, the Scikit-learn [118] (version 0.24.2) library implementation of the GaussianProcessRegressor class was used with the kernel combination of the sum of WhiteKernel, DotProduct and the RBF kernel from the same library module. The fitting of the GaussianProcessRegressor also optimized the kernel’s hyperparameters [118]. Of the GPR models with the same number of descriptors, the model with the highest coefficient of determination was considered optimal. Whether a higher parameter model was better than a model with one less parameter was based on a 20% error improvement, similarly to the SVR descriptor selection method.

### 4.6. Diagnostics and Applicability Domain of Models

The performance of models was evaluated using 10-fold cross-validation to avoid overfitting. The model predictive capability was assessed in the training process by calculating the coefficient of determination (Equation (10)), $r^{2}$, and evaluation external to descriptor selection and producing the models was measured by the concordance correlation coefficient (Equation (11)) [119], CCC. As another comparison tool, the mean squared error (Equation (12)), RMSE, has been provided along with the models’ statistical parameters.

(10) $$r^{2}=1-\frac{\sum {\left(y_{i}-{\hat{y}}_{i}\right)}^{2}}{\sum {\left(y_{i}-{\overset{-}{y}}_{i}\right)}^{2}},$$

(11) $$CCC=\frac{2\sum_{i=1}^{n}\left({\hat{y}}_{i}-\overset{-}{\hat{y}}\right){\left(y_{i}-\overset{-}{y}\right)}_{}}{\sum_{i=1}^{n}{\left({\hat{y}}_{i}-\overset{-}{\hat{y}}\right)}^{2}+\sum_{i=1}^{n}{\left(y_{i}-\overset{-}{y}\right)}^{2}+n{\left(\overset{-}{\hat{y}}-\hat{y}\right)}^{2}},$$

(12) $$RMSE=\sqrt{\frac{1}{n}\sum {\left(y_{i}-{\hat{y}}_{i}\right)}^{2}}$$

Additional diagnostics were performed for the linear models to identify outliers, high leverage data points, and influential data points. Data points exceeding the critical leverage value $h^{*}{}_{3}$ (Equation (14)) are considered as high leverage, where the critical value is calculated from the model’s descriptor amount k and data set size n. A matrix of model molecular descriptor values in columns along with an additional constant column comprises design matrix X used in the calculation of the leverage $h_{ii}$ of a data point i:

(13) $$H=X\cdot {\left(X^{T}\cdot X\right)}^{-1}\cdot X^{T},$$

(14) $$h^{*}{}_{3}=\frac{3\cdot \left(k+1\right)}{n},$$

where *H* is called the hat matrix and $h_{ii}$ includes its main diagonal values. For outlier diagnostics, the standardized residuals $r_{i}$ of the model are examined for each data point i:

(15) $$r_{i}=\frac{{\hat{y}}_{i}-y_{i}}{\hat{\sigma }\cdot \sqrt{1-h_{ii}}},$$

(16) $${\hat{\sigma }}^{2}=\frac{e^{T}\cdot e}{n-k-1},$$

(17) $$e_{i}=\left(1-h_{ii}\right)\cdot y_{i},$$

where $\hat{\sigma }$ is the mean squared error of the linear regression model, and $e_{i}$ is the residual of the i-th data point. A data point with $\left|r_{i}\right|>2$ should be inspected and a data point with $\left|r_{i}\right|>3$ is likely to be an outlier and requires closer analysis. 

An observation’s influence on the model is assessed by Cook’s distance (*D_i_*), a measure of the effect of removing a given data point.

(18) $$D_{i}=\frac{r_{i}{}^{2}}{k+1}\cdot \frac{h_{ii}}{1-h_{ii}}$$

A common rule is to take a closer look at observations with Cook’s distance higher than one. The accuracy of the model’s predictions may be distorted by high leverage and/or high residual observations and Cook’s distance provides a method to find influential data points that could indicate the regions of the molecular space, where more experimental data are required. 

### 4.7. Availability of Regression Models

The MLR, SVR and GPR models and related data can be made available in various data formats [120]. To follow the best practices of QSAR model reporting [121], the models with data are stored at the QsarDB repository [122] in QSAR Data Bank format [123]. A digital object identifier (DOI) has been assigned for the models and data [124].

## 5. Conclusions

The present study successfully tested the hypothesis that the gas-ionic liquid partition coefficient (log *K*) can be successfully modeled in a data-driven manner based on the partial structure of the ionic liquid, namely the varying cationic counterpart. QSPR models were derived for gas-ionic liquid partition coefficients for three organic compounds hexane, cyclohexane and benzene in the series of ionic liquids with common bis(trifluoromethylsulfonyl)imide ([Tf2N]^−^) anion using linear and non-linear QSPR methods. Variation in ionic counterpart makes it possible to more specifically understand the molecular interactions of partitioning by the ionic liquid and how to enable finding the application-appropriate ionic liquid. Three machine learning approaches (MLR, SVR and GPR) were used to derive data driven models and their performance was compared. The comparison of different modeling methods showed that both linear and non-linear models have excellent performance, while non-linear models had better performance. The selection of a suitable model for the prediction of log *K* depends on the circumstances and they offer different benefits. For example, the MLR models have an advantage in that they are simple and easy to interpret. The SVM models had the best prediction performance, and GPR models can provide uncertainty measurements on the predictions. The cross-validation coefficients of determination were in the range of 0.71–0.93 and also other performance statistics indicated strong accuracy of models for all data series and machine learning methods. The analysis and interpretation of descriptors revealed how the structure of cationic counterpart influences molecular interactions and that generally higher lipophilicity and dispersion interaction capability and lower polarity in the cations induces a higher partition coefficient for benzene, hexane, cyclohexane and hydrocarbons in general. Applicability domain analysis of models exposed outliers, but it concluded that they are not highly influential and that the models are applicable to a wide selection of cation families with variable size, polarity and aliphatic or aromatic nature.

## Figures and Tables

**Table 1:** Hyperparameters of the SVR and GPR final models.

	C	ε	γ
SVRh	1	0.001	auto
SVRc	5	0.001	0.1
SVRb	1	0.001	scale
	**Sigma_0**	**Noise_Level**	**Length_Scale**
GPRh	0.478	0.00947	3.7
GPRc	0.364	0.00888	9.52
GPRb	3.13	0.00215	2.91

**Table 2:** Statistical parameters of final linear and non-linear models on all cross-validation folds.

	R^2^			RMSE			CCC		
	**MLRh**	**SVRh**	**GPRh**	**MLRh**	**SVRh**	**GPRh**	**MLRh**	**SVRh**	**GPRh**
train	0.944	0.966	0.957	0.092	0.071	0.080	0.971	0.982	0.978
test	0.919	0.926	0.924	0.101	0.098	0.097	0.960	0.957	0.957
	**MLRc**	**SVRc**	**GPRc**	**MLRc**	**SVRc**	**GPRc**	**MLRc**	**SVRc**	**GPRc**
train	0.915	0.946	0.940	0.102	0.081	0.085	0.955	0.972	0.969
test	0.891	0.910	0.903	0.110	0.097	0.097	0.942	0.953	0.950
	**MLRb**	**SVRb**	**GPRb**	**MLRb**	**SVRb**	**GPRb**	**MLRb**	**SVRb**	**GPRb**
train	0.791	0.973	0.935	0.068	0.025	0.038	0.883	0.986	0.966
test	0.717	0.869	0.788	0.072	0.051	0.057	0.813	0.928	0.869

**Table 3:** Standardized regression coefficients of descriptors for linear models and permutation importance of descriptors for linear and non-linear models. Between the models, the columns are attributed to the same or similar descriptor where possible.

Model	Descriptors: Standardized Regression Coefficients					
MLRh	*VE2_A*	*AATS7s*	*ATSC0s*	*AATSC2dv*	*Xpc-4d*	
	−0.326	−0.089	−0.126	0.060	−0.133	
MLRc	*VE2_A*	*GATS7Z*	*ATSC0s*		*Xpc-4d*	
	−0.329	0.101	−0.153		−0.126	
MLRb		*GATS3m*	*AATS0s*	*GATS2dv*		
		−0.042	−0.131	−0.055		
**Permutation Importances**						
MLRh	*VE2_A*	*AATS7s*	*ATSC0s*	*AATSC2dv*	*Xpc−4d*	
	1.43	0.096	0.214	0.048	0.237	
MLRc	*VE2_A*	*GATS7Z*	*ATSC0s*		*Xpc-4d*	
	1.79	0.156	0.376		0.264	
MLRb		*GATS3m*	*AATS0s*	*GATS2dv*		
		1.50	0.272	0.166		
SVRh	*SMR_VSA5*	*AATS6m*	*AATSC0s*		*Xc-5d*	*SpMAD_D*
	0.324	0.155	0.422		0.189	0.351
SVRc	*SMR_VSA5*	*AATS7m*	*AATSC0s*		*Xc-5d*	
	1.04	0.0938	0.226		0.237	
SVRb	*Mi*	*AATSC8i*	*ATSC1s*	*GATS2pe*		
	0.837	0.218	0.511	0.319		
GPRh	*SMR_VSA5*		*GATS1s*	*ATSC1are*	*Xpc-4d*	
	1.92		0.0472	0.476	0.564	
GPRc	*SLogP*	*MATS8c*	*AATS0s*	*AATSC6se*	*Xpc-4dv*	
	1.33	0.0664	0.254	0.0129	0.513	
GPRb	*MDEC-12*	*GATS3Z*	*AATSC0s*			
	0.638	0.985	1.01			

**Table 4:** Descriptor structural contribution and related solvent interaction based on descriptor analysis.

Solvent Interaction	Main Structural Contribution	Descriptors		
		MLR	SVR	GPR
**Dispersion Forces** (molecule size, polarizability and molecule shape)	Atom count/chain length	*VE2_A*, *GATS3m*	*SpMAD_D*, *Mi* *	*MDEC-12*, *GATS3Z*
	Molecule surface area		*SMR_VSA5*	*SMR_VSA5*
	Branching	*Xpc-4d*, *AATSC2dv*, *GATS2dv*	*Xc-5d*	*Xpc-4d*, *Xpc-4dv*
	Lipophilicity			*SLogP*
**Coulomb and Dipolar Interactions** (Charge/electron cloud distribution)				
	Gasteiger charge			*MATS8c*
	Electronegativity		*Mi* *, *AATSC8i*, *GATS2pe*	*ATSC1are*, *AATSC6se*
	Bond order	*AATSC2dv*, *GATS2dv*		
	Heteroatoms/hydrogen bonding atoms	*AATS7s*, *AATS0s*, *ATSC0s*, *GATS7Z*, *ATSC0s*	*AATSC0s*, *AATS6m*, *AATS7m*, *AATSC8i* *ATSC1s*	*GATS1s*, *AATS0s*, *AATSC6se*, *AATSC0s*
**Hydrogen Bonding** (Presence of HB capable hetero atoms)				

* descriptors that relate to multiple structural contributions.

**Table 5:** Structures and abbreviations of cations.

[PrOHMMorp]^+^ 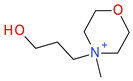	[4-CNBPy]^+^ 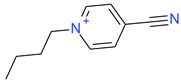	[EtOHMIm]^+^ 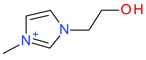
[EtOHM_3_Am]^+^ 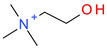	[Et_3_S]^+^ 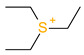	[1-PrOHPy]^+^ 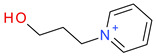
[CNMeM_2_iPAm]^+^ 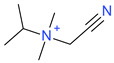	[(Meo)_2_Im]^+^ 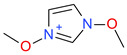	[M_3_BAm]^+^ 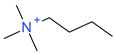
[BzPy]^+^ 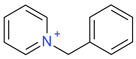	[MeoeMMorp]^+^ 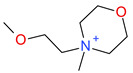	[EtOHM_2_iPAm]^+^ 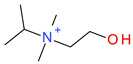
[TDC]^+^ 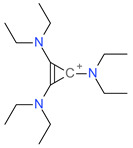	[C_1,9_(M_2_iPAm)_2_]^2+^ 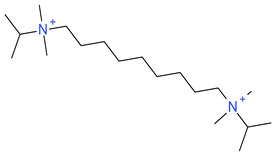	[BzMPyrr]^+^ 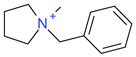

**Table 6:** SVR hyperparameter tuning values.

C	0.001, 0.005, 0.1, 0.5, 1,5, 10, 50, 100, 500, 1000
ε	0.001, 0.005, 0.01, 0.05, 0.1, 0.5, 1.0, 5.0, 10.0
γ	0.001, 0.005, 0.01, 0.05, 0.1, ‘auto’, ‘scale’

## Data Availability

The data and model presented in this study are openly available at QsarDB Repository at http://dx.doi.org/10.15152/QDB.256, accessed on 6 July 2022.

## Supplementary Materials

The following supporting information can be downloaded at: https://www.mdpi.com/article/10.3390/ijms23147534/s1.
