# Supplementary material for: Machine Learning Quantitative Structure–Property Relationships as a Function of Ionic Liquid Cations for the Gas-Ionic Liquid Partition Coefficient of Hydrocarbons
Source: Int J Mol Sci. 2022 Jul 7;23(14):7534. doi: 10.3390/ijms23147534 (PMC9323540; doi:10.3390/ijms23147534)

Machine learning Quantitative Structure-Property Relationships as a function of ionic liquid cations for the gas-ionic liquid partition coefficient of hydrocarbons

Karl Marti Toots[1], Sulev Sild[1], Jaan Leis[1], William E. Acree Jr.[2], Uko Maran[1*]

[1] Department of Chemistry, University of Tartu, 14a Ravila Street, Tartu 50411, Estonia
[2] Department of Chemistry, University of North Texas, 1155 Union Circle Drive #305070, Denton, TX, 76203-5017, USA
* Corresponding author

Supplementary

Table S1. Experimental and predicted values of the MLRh model containing the training and validation fold values. Experimental values pertain to 298.15 K, and were retrieved from the published literature by William E. Acree.[42,70–106]

		Fold 1	Fold 2	Fold 3	Fold 4	Fold 5	Fold 6	Fold 7	Fold 8	Fold 9	Fold 10	Exp.
([PrOHMMorp]+[(Tf)2N]-)	1	0.572	0.437	0.469	0.463	0.406	0.448	0.473	0.451	0.471	0.459	0.209
([EtOHMIM]+[Tf2N]-)	2	0.657	0.554	0.576	0.581	0.528	0.566	0.585	0.565	0.580	0.568	0.623
([EtOHM3Am]+[(Tf)2N]-)	3	0.782	0.768	0.775	0.805	0.784	0.850	0.799	0.791	0.779	0.792	0.790
[(Meo)2Im]+[Tf2N]-)	4	0.914	0.859	0.870	0.869	0.847	0.863	0.876	0.869	0.878	0.848	0.811
([1-PrOHPy]+[(Tf)2N]-)	5	0.785	0.680	0.700	0.709	0.649	0.683	0.708	0.688	0.705	0.691	0.904
([C1,9(M2iPAm)2]+[(Tf)2N]-)	6	1.008	0.980	1.013	0.952	1.010	1.037	0.988	1.002	0.996	1.017	0.932
([MeoeMMorp]+[(Tf)2N]-)	7	1.061	1.020	1.033	1.031	1.024	1.043	1.038	1.038	1.041	1.010	0.962
([4-CNBPy]+[(Tf)2N]-)	8	1.083	1.007	1.026	1.031	0.979	1.024	1.022	1.006	1.016	1.057	1.064
([BzMIm]+[Tf2N]-)	9	1.241	1.200	1.209	1.201	1.191	1.189	1.206	1.208	1.217	1.180	1.106
([MeoeMIm]+[Tf2N]-)	10	1.131	1.089	1.097	1.099	1.084	1.090	1.102	1.101	1.109	1.067	1.108
([BzPy]+[Tf2N]-)	11	1.271	1.232	1.239	1.236	1.223	1.219	1.238	1.240	1.249	1.206	1.115
([PM2iPAm]+[(Tf)2N]-)	12	1.026	1.028	1.039	1.019	1.046	1.069	1.034	1.042	1.035	1.037	1.118
([BzMPyrr]+[Tf2N]-)	13	1.358	1.347	1.355	1.343	1.351	1.358	1.347	1.354	1.355	1.345	1.129
([NEP]+[Tf2N]-)	14	1.208	1.221	1.217	1.228	1.223	1.238	1.223	1.225	1.221	1.214	1.133
([PMPip]+[(Tf)2N]-)	15	1.308	1.315	1.317	1.313	1.322	1.332	1.314	1.322	1.318	1.311	1.156
([Et3S]+[(Tf)2N]-)	16	1.316	1.370	1.360	1.379	1.389	1.417	1.369	1.377	1.361	1.373	1.195
([tert-BMIm]+[Tf2N]-)	17	1.138	1.144	1.152	1.139	1.157	1.181	1.147	1.154	1.147	1.157	1.202
([M2EIm]+[Tf2N]-)	18	1.228	1.244	1.246	1.241	1.252	1.274	1.242	1.248	1.241	1.259	1.210
([MEIm]+[Tf2N]-)	19	1.201	1.215	1.212	1.219	1.218	1.236	1.216	1.219	1.213	1.214	1.242
([AllMIm]+[(Tf)2N]-)	20	1.140	1.112	1.117	1.118	1.103	1.112	1.119	1.117	1.122	1.103	1.266
([MeoeE2MAm]+[(Tf)2N]-)	21	1.119	1.096	1.107	1.099	1.102	1.121	1.106	1.109	1.109	1.097	1.294
([MeoeMPip]+[(Tf)2N]-)	22	1.225	1.205	1.213	1.209	1.209	1.222	1.213	1.217	1.218	1.198	1.318
([M3BAm]+[Tf2N]-)	23	1.246	1.255	1.253	1.260	1.260	1.272	1.258	1.262	1.259	1.241	1.330
([C3MPyrr]+[Tf2N]-)	24	1.248	1.261	1.262	1.259	1.271	1.284	1.262	1.269	1.263	1.258	1.334
([sec-BMIm]+[Tf2N]-)	25	1.304	1.309	1.312	1.308	1.312	1.329	1.307	1.312	1.309	1.319	1.370
([HexM3Am]+[Tf2N]-)	26	1.438	1.434	1.434	1.437	1.432	1.434	1.432	1.439	1.440	1.419	1.390
([BMPyrr]+[(Tf)2N]-)	27	1.353	1.361	1.363	1.358	1.368	1.377	1.359	1.368	1.364	1.356	1.393
([C5MPyrr]+[Tf2N]-)	28	1.434	1.432	1.436	1.429	1.436	1.439	1.429	1.438	1.438	1.427	1.407
([4-MBPy]+[Tf2N]-)	29	1.448	1.446	1.447	1.448	1.441	1.448	1.442	1.447	1.448	1.445	1.417
([BMPip]+[(Tf)2N]-)	30	1.410	1.416	1.418	1.413	1.422	1.429	1.413	1.422	1.420	1.411	1.419
([ChxPyr]+[Tf2N]-)	31	1.447	1.437	1.439	1.440	1.432	1.434	1.435	1.440	1.443	1.428	1.432
([MBIm]+[Tf2N]-)	32	1.438	1.439	1.439	1.442	1.434	1.443	1.435	1.439	1.439	1.440	1.435
([ChxMIm]+[Tf2N]-)	33	1.422	1.411	1.415	1.411	1.408	1.411	1.409	1.414	1.417	1.406	1.447
([HM2iPAm]+[(Tf)2N]-)	34	1.289	1.278	1.292	1.267	1.290	1.301	1.279	1.289	1.288	1.286	1.462
([ChxMPyrr]+[Tf2N]-)	35	1.449	1.455	1.459	1.449	1.464	1.469	1.451	1.462	1.460	1.451	1.519
([C6MPyrr]+[Tf2N]-)	36	1.547	1.549	1.553	1.546	1.554	1.555	1.544	1.555	1.554	1.544	1.522
([PeMPip]+[(Tf)2N]-)	37	1.482	1.482	1.486	1.478	1.485	1.488	1.478	1.487	1.487	1.476	1.549
([MB3Am]+[(Tf)2N]-)	38	1.657	1.662	1.666	1.660	1.668	1.671	1.655	1.667	1.665	1.662	1.586
([Quin6]+[(Tf)2N]-)	39	1.561	1.565	1.571	1.557	1.574	1.576	1.559	1.571	1.570	1.565	1.601

([TDC]+[Tf2N]-)	40	1.729	1.727	1.739	1.717	1.728	1.741	1.713	1.725	1.724	1.763	1.606
([HexomMIm]+[Tf2N]-)	41	1.657	1.634	1.640	1.643	1.625	1.630	1.631	1.636	1.642	1.637	1.622
([C8MPyrr]+[Tf2N]-)	42	1.725	1.730	1.734	1.727	1.736	1.733	1.721	1.735	1.735	1.725	1.630
([HMPip]+[(Tf)2N]-)	43	1.581	1.584	1.588	1.581	1.589	1.589	1.578	1.589	1.589	1.579	1.641
([MHIm]+[Tf2N]-)	44	1.625	1.622	1.623	1.624	1.615	1.617	1.614	1.621	1.623	1.622	1.660
([M3OAm]+[(Tf)2N]-)	45	1.647	1.647	1.647	1.650	1.647	1.645	1.641	1.651	1.652	1.633	1.672
([OE3Am]+[(Tf)2N]-)	46	1.708	1.714	1.712	1.718	1.712	1.710	1.706	1.716	1.717	1.703	1.748
([DM3Am]+[(Tf)2N]-)	47	1.809	1.811	1.811	1.814	1.812	1.806	1.802	1.815	1.816	1.799	1.783
([MOIm]+[Tf2N]-)	48	1.799	1.799	1.800	1.802	1.795	1.793	1.789	1.799	1.801	1.798	1.793
([C10MPyrr]+[Tf2N]-)	49	1.862	1.868	1.872	1.865	1.874	1.869	1.857	1.872	1.872	1.863	1.810
([Quin8]+[(Tf)2N]-)	50	1.710	1.716	1.722	1.708	1.725	1.724	1.706	1.722	1.720	1.716	1.838
([OiQu]+[(Tf)2N]-)	51	1.846	1.845	1.850	1.844	1.841	1.844	1.832	1.843	1.844	1.860	1.839
[(Hexom)2Im]+[Tf2N]-)	52	1.868	1.836	1.846	1.851	1.825	1.836	1.832	1.837	1.844	1.855	1.918
([DoMIm]+[(Tf)2N]-)	53	2.050	2.055	2.056	2.059	2.053	2.047	2.040	2.054	2.056	2.055	2.002
([D2MIm]+[(Tf)2N]-)	54	2.126	2.128	2.134	2.127	2.130	2.128	2.112	2.129	2.130	2.140	2.074
([O4P]+[(Tf)2N]-)	55	2.179	2.181	2.188	2.183	2.188	2.184	2.167	2.186	2.186	2.189	2.168
([H3TdP]+[(Tf)2N]-)	56	2.189	2.191	2.198	2.193	2.198	2.193	2.176	2.195	2.196	2.199	2.192
([MO3Am]+[(Tf)2N]-)	57	2.089	2.092	2.098	2.092	2.098	2.092	2.078	2.096	2.097	2.094	2.248

Table S2. Experimental and predicted values of the MLRc model containing the training and validation fold values. Experimental values pertain to 298.15 K, and were retrieved from the published literature by William E. Acree.[42,70–106]

		Fold 1	Fold 2	Fold 3	Fold 4	Fold 5	Fold 6	Fold 7	Fold 8	Fold 9	Fold 10	Exp.
([PrOHMMorp]+[(Tf)2N]-)	1	1.255	1.183	1.193	1.156	1.170	1.160	1.183	1.180	1.193	1.158	0.836
([CNMeM2iPAm]+[(Tf)2N]-)	2	1.169	1.114	1.095	1.085	1.102	1.099	1.131	1.099	1.126	1.093	1.121
[(Meo)2Im]+[Tf2N]-)	3	1.329	1.294	1.332	1.299	1.297	1.278	1.296	1.300	1.307	1.292	1.226
([EtOHM2iPAm]+[(Tf)2N]-)	4	1.180	1.126	1.108	1.098	1.115	1.112	1.144	1.111	1.138	1.106	1.228
([EtOHMIM]+[Tf2N]-)	5	1.226	1.164	1.186	1.153	1.156	1.146	1.157	1.158	1.177	1.149	1.235
([EtOHM3Am]+[(Tf)2N]-)	6	1.231	1.179	1.180	1.176	1.171	1.174	1.171	1.150	1.194	1.171	1.280
([C1,9(M2iPAm)2]+[(Tf)2N]-)	7	1.464	1.447	1.427	1.408	1.446	1.423	1.511	1.475	1.451	1.426	1.381
([1-PrOHPy]+[(Tf)2N]-)	8	1.374	1.315	1.334	1.306	1.305	1.300	1.303	1.304	1.325	1.299	1.399
([MeoeMMorp]+[(Tf)2N]-)	9	1.671	1.649	1.648	1.650	1.645	1.646	1.656	1.637	1.657	1.644	1.434
([4-CNBPy]+[(Tf)2N]-)	10	1.449	1.387	1.398	1.369	1.375	1.370	1.382	1.381	1.395	1.366	1.516
([PM2iPAm]+[(Tf)2N]-)	11	1.585	1.588	1.574	1.594	1.591	1.591	1.615	1.575	1.598	1.592	1.526
([MeoeMIm]+[Tf2N]-)	12	1.653	1.634	1.654	1.645	1.635	1.629	1.634	1.629	1.643	1.634	1.528
([BzPy]+[Tf2N]-)	13	1.792	1.783	1.801	1.797	1.785	1.781	1.785	1.779	1.790	1.785	1.557
([NEP]+[Tf2N]-)	14	1.703	1.708	1.711	1.738	1.712	1.721	1.705	1.678	1.720	1.723	1.589
([BzMIm]+[Tf2N]-)	15	1.727	1.715	1.735	1.724	1.717	1.708	1.722	1.717	1.722	1.714	1.606
([PMPip]+[(Tf)2N]-)	16	1.745	1.748	1.752	1.765	1.752	1.752	1.759	1.736	1.757	1.755	1.623
([M2EIm]+[Tf2N]-)	17	1.680	1.684	1.676	1.700	1.686	1.692	1.697	1.662	1.694	1.692	1.624
([Et3S]+[(Tf)2N]-)	18	1.633	1.638	1.644	1.669	1.643	1.650	1.637	1.609	1.651	1.654	1.637
([BzMPyrr]+[Tf2N]-)	19	1.768	1.762	1.769	1.768	1.763	1.758	1.777	1.762	1.768	1.761	1.648
([tert-BMIm]+[Tf2N]-)	20	1.654	1.656	1.642	1.664	1.657	1.661	1.676	1.638	1.665	1.660	1.672
([MEIm]+[Tf2N]-)	21	1.681	1.686	1.686	1.712	1.689	1.697	1.687	1.658	1.697	1.699	1.676
([AllMIm]+[(Tf)2N]-)	22	1.588	1.576	1.597	1.593	1.579	1.573	1.576	1.568	1.586	1.581	1.702
([MeoeE2MAm]+[(Tf)2N]-)	23	1.604	1.589	1.593	1.591	1.588	1.583	1.603	1.584	1.597	1.586	1.714
([C3MPyrr]+[Tf2N]-)	24	1.777	1.786	1.779	1.807	1.789	1.798	1.797	1.763	1.796	1.797	1.716
([sec-BMIm]+[Tf2N]-)	25	1.699	1.696	1.699	1.710	1.698	1.699	1.706	1.683	1.705	1.701	1.729
([BM2Im]+[(Tf)2N]-)	26	1.694	1.686	1.699	1.694	1.689	1.680	1.700	1.687	1.694	1.686	1.734
([HexM3Am]+[Tf2N]-)	27	1.835	1.832	1.847	1.850	1.834	1.833	1.833	1.824	1.838	1.836	1.735
([MeoeMPip]+[(Tf)2N]-)	28	1.721	1.709	1.714	1.716	1.709	1.707	1.718	1.701	1.716	1.708	1.752
([BMPyrr]+[(Tf)2N]-)	29	1.785	1.788	1.791	1.804	1.791	1.792	1.799	1.777	1.796	1.794	1.764
([HM2iPAm]+[(Tf)2N]-)	30	1.697	1.690	1.691	1.686	1.691	1.681	1.718	1.698	1.696	1.685	1.803
([BMPip]+[(Tf)2N]-)	31	1.833	1.836	1.838	1.850	1.838	1.839	1.847	1.826	1.842	1.840	1.832
([MBIm]+[Tf2N]-)	32	1.756	1.750	1.768	1.769	1.753	1.750	1.752	1.743	1.759	1.755	1.845
([4-MBPy]+[Tf2N]-)	33	1.800	1.795	1.809	1.811	1.796	1.795	1.797	1.787	1.802	1.799	1.846
([C6MPyrr]+[Tf2N]-)	34	1.920	1.920	1.926	1.931	1.921	1.920	1.931	1.918	1.924	1.921	1.880
([C5MPyrr]+[Tf2N]-)	35	1.783	1.781	1.795	1.792	1.784	1.777	1.792	1.781	1.787	1.783	1.881
([ChxMIm]+[Tf2N]-)	36	1.764	1.755	1.774	1.767	1.758	1.750	1.763	1.756	1.762	1.756	1.890
([ChxPyr]+[Tf2N]-)	37	1.822	1.816	1.834	1.832	1.818	1.815	1.819	1.812	1.823	1.820	1.898
([PeMPip]+[(Tf)2N]-)	38	1.835	1.833	1.845	1.844	1.836	1.831	1.845	1.834	1.839	1.835	1.916
([ChxMPyrr]+[Tf2N]-)	39	1.843	1.845	1.849	1.855	1.847	1.845	1.861	1.842	1.851	1.847	1.945
([MB3Am]+[(Tf)2N]-)	40	2.070	2.072	2.065	2.080	2.069	2.076	2.082	2.063	2.073	2.070	1.959
([Quin6]+[(Tf)2N]-)	41	1.929	1.931	1.932	1.936	1.931	1.929	1.949	1.932	1.934	1.929	1.978
([HexomMIm]+[Tf2N]-)	42	2.027	2.011	2.017	2.015	2.005	2.008	2.010	2.006	2.012	2.003	2.000
([MHIm]+[Tf2N]-)	43	1.969	1.965	1.975	1.980	1.965	1.967	1.967	1.958	1.969	1.966	2.002
([M3OAm]+[(Tf)2N]-)	44	2.039	2.038	2.046	2.054	2.037	2.042	2.040	2.030	2.041	2.040	2.010
([HMPip]+[(Tf)2N]-)	45	1.954	1.955	1.960	1.965	1.955	1.955	1.966	1.952	1.958	1.955	2.019
([TDC]+[Tf2N]-)	46	1.926	1.912	1.911	1.900	1.908	1.900	1.935	1.925	1.912	1.898	2.029
([C8MPyrr]+[Tf2N]-)	47	2.102	2.105	2.104	2.113	2.103	2.107	2.116	2.102	2.106	2.103	2.040
([M3BAm]+[Tf2N]-)	48	1.796	1.803	1.801	1.830	1.806	1.817	1.804	1.775	1.813	1.816	2.059
([OE3Am]+[(Tf)2N]-)	49	2.063	2.063	2.072	2.081	2.063	2.068	2.062	2.054	2.066	2.066	2.096

([OiQu]+[(Tf)2N]-)	50	2.159	2.154	2.150	2.157	2.149	2.155	2.163	2.151	2.153	2.147	2.133
([C10MPyrr]+[Tf2N]-)	51	2.242	2.246	2.239	2.251	2.242	2.249	2.257	2.243	2.244	2.241	2.150
([MOIm]+[Tf2N]-)	52	2.154	2.152	2.154	2.164	2.149	2.156	2.154	2.144	2.153	2.150	2.153
([Quin8]+[(Tf)2N]-)	53	2.084	2.087	2.082	2.090	2.086	2.087	2.105	2.088	2.087	2.083	2.187
([DM3Am]+[(Tf)2N]-)	54	2.199	2.200	2.201	2.213	2.197	2.205	2.201	2.192	2.200	2.198	2.191
[(Hexom)2Im]+[Tf2N]-)	55	2.232	2.205	2.196	2.195	2.190	2.201	2.202	2.199	2.200	2.185	2.253
([DoMIm]+[(Tf)2N]-)	56	2.404	2.405	2.396	2.412	2.397	2.411	2.406	2.396	2.400	2.398	2.321
([D2MIm]+[(Tf)2N]-)	57	2.469	2.467	2.447	2.461	2.456	2.469	2.477	2.464	2.459	2.452	2.396
([O4P]+[(Tf)2N]-)	58	2.505	2.499	2.481	2.491	2.488	2.499	2.509	2.500	2.490	2.482	2.488
([H3TdP]+[(Tf)2N]-)	59	2.503	2.497	2.480	2.488	2.486	2.497	2.507	2.499	2.488	2.480	2.531
([MO3Am]+[(Tf)2N]-)	60	2.462	2.462	2.444	2.459	2.452	2.465	2.471	2.459	2.454	2.449	2.569

Table S3. Experimental and predicted values of the MLRb model containing the training and validation fold values. Experimental values pertain to 298.15 K, and were retrieved from the published literature by William E. Acree.[42,70–106]

		Fold 1	Fold 2	Fold 3	Fold 4	Fold 5	Fold 6	Fold 7	Fold 8	Fold 9	Fold 10	Exp.
[(Meo)2Im]+[Tf2N]-)	1	2.518	2.503	2.520	2.512	2.489	2.479	2.515	2.507	2.506	2.510	2.395
([EtOHMIM]+[Tf2N]-)	2	2.476	2.454	2.481	2.469	2.447	2.432	2.474	2.463	2.467	2.466	2.401
([PrOHMMorp]+[(Tf)2N]-)	3	2.579	2.566	2.584	2.575	2.559	2.552	2.578	2.574	2.576	2.574	2.434
([EtOHM2iPAm]+[(Tf)2N]-)	4	2.529	2.518	2.532	2.525	2.502	2.506	2.525	2.530	2.526	2.524	2.468
([EtOHM3Am]+[(Tf)2N]-)	5	2.419	2.403	2.422	2.414	2.380	2.389	2.414	2.419	2.413	2.412	2.517
([CNMeM2iPAm]+[(Tf)2N]-)	6	2.499	2.486	2.500	2.493	2.466	2.461	2.495	2.489	2.485	2.491	2.530
([BzPy]+[Tf2N]-)	7	2.772	2.771	2.775	2.771	2.770	2.754	2.774	2.765	2.768	2.772	2.580
([C1,9(M2iPAm)2]+[(Tf)2N]-)	8	2.734	2.740	2.739	2.736	2.728	2.746	2.733	2.752	2.746	2.740	2.583
([HexM3Am]+[Tf2N]-)	9	2.716	2.720	2.721	2.718	2.708	2.729	2.714	2.735	2.729	2.721	2.608
([MeoeMIm]+[Tf2N]-)	10	2.671	2.667	2.674	2.669	2.659	2.656	2.670	2.671	2.670	2.670	2.633
([PM2iPAm]+[(Tf)2N]-)	11	2.675	2.678	2.679	2.676	2.662	2.687	2.672	2.696	2.688	2.680	2.640
([1-PrOHPy]+[(Tf)2N]-)	12	2.581	2.562	2.586	2.575	2.563	2.540	2.581	2.564	2.573	2.573	2.663
([MeoeMMorp]+[(Tf)2N]-)	13	2.682	2.680	2.685	2.681	2.670	2.673	2.681	2.686	2.684	2.682	2.691
([M3BAm]+[Tf2N]-)	14	2.650	2.653	2.655	2.652	2.635	2.664	2.646	2.673	2.664	2.655	2.696
([BzMIm]+[Tf2N]-)	15	2.743	2.742	2.745	2.741	2.737	2.727	2.743	2.739	2.739	2.742	2.715
([tert-BMIm]+[Tf2N]-)	16	2.678	2.683	2.677	2.677	2.660	2.667	2.675	2.682	2.671	2.678	2.719
([HM2iPAm]+[(Tf)2N]-)	17	2.749	2.754	2.754	2.751	2.745	2.760	2.748	2.765	2.761	2.754	2.730
([BzMPyrr]+[Tf2N]-)	18	2.829	2.836	2.830	2.829	2.830	2.823	2.830	2.829	2.827	2.832	2.754
([M3OAm]+[(Tf)2N]-)	19	2.759	2.765	2.764	2.762	2.756	2.771	2.759	2.776	2.772	2.765	2.792
([MB3Am]+[(Tf)2N]-)	20	2.846	2.853	2.851	2.848	2.853	2.855	2.848	2.856	2.857	2.852	2.796
([NEP]+[Tf2N]-)	21	2.855	2.858	2.858	2.854	2.861	2.841	2.858	2.847	2.852	2.856	2.796
([sec-BMIm]+[Tf2N]-)	22	2.834	2.839	2.836	2.834	2.837	2.824	2.836	2.831	2.831	2.836	2.799
([C6MPyrr]+[Tf2N]-)	23	2.848	2.857	2.852	2.850	2.854	2.857	2.849	2.858	2.857	2.854	2.800
([DM3Am]+[(Tf)2N]-)	24	2.790	2.797	2.795	2.793	2.791	2.802	2.791	2.805	2.802	2.797	2.803
([MeoeE2MAm]+[(Tf)2N]-)	25	2.717	2.718	2.721	2.717	2.709	2.717	2.716	2.726	2.724	2.719	2.808
([4-CNBPy]+[(Tf)2N]-)	26	2.675	2.660	2.679	2.669	2.666	2.628	2.678	2.649	2.660	2.667	2.811
([MEIm]+[Tf2N]-)	27	2.797	2.801	2.799	2.797	2.796	2.786	2.799	2.794	2.794	2.798	2.812
([M2EIm]+[Tf2N]-)	28	2.801	2.805	2.802	2.800	2.799	2.790	2.802	2.799	2.797	2.802	2.814
([Et3S]+[(Tf)2N]-)	29	2.808	2.829	2.801	2.809	2.796	2.796	2.807	2.807	2.789	2.810	2.823
([BM2Im]+[(Tf)2N]-)	30	2.859	2.865	2.862	2.859	2.865	2.852	2.862	2.857	2.858	2.862	2.828
([AllMIm]+[(Tf)2N]-)	31	2.707	2.704	2.710	2.705	2.698	2.690	2.707	2.704	2.704	2.706	2.833
([O4P]+[(Tf)2N]-)	32	2.922	2.935	2.923	2.924	2.933	2.924	2.925	2.924	2.923	2.927	2.841
([ChxMIm]+[Tf2N]-)	33	2.894	2.902	2.896	2.895	2.903	2.889	2.897	2.892	2.894	2.898	2.842
([HexomMIm]+[Tf2N]-)	34	2.834	2.836	2.838	2.834	2.840	2.825	2.837	2.831	2.835	2.836	2.845
[(Hexom)2Im]+[Tf2N]-)	35	2.870	2.873	2.875	2.871	2.881	2.862	2.874	2.866	2.872	2.873	2.847
([BMPyrr]+[(Tf)2N]-)	36	2.825	2.834	2.829	2.827	2.828	2.834	2.826	2.836	2.834	2.831	2.849
([MeoeMPip]+[(Tf)2N]-)	37	2.775	2.778	2.779	2.775	2.773	2.775	2.775	2.782	2.780	2.778	2.850
([PMPip]+[(Tf)2N]-)	38	2.832	2.840	2.836	2.834	2.836	2.841	2.833	2.843	2.841	2.838	2.853
([H3TdP]+[(Tf)2N]-)	39	2.922	2.935	2.923	2.924	2.933	2.924	2.925	2.924	2.923	2.927	2.862
([C10MPyrr]+[Tf2N]-)	40	2.878	2.887	2.882	2.880	2.887	2.886	2.880	2.886	2.886	2.884	2.880
([C3MPyrr]+[Tf2N]-)	41	2.810	2.818	2.814	2.812	2.811	2.819	2.811	2.822	2.819	2.816	2.885
([MHIm]+[Tf2N]-)	42	2.900	2.907	2.903	2.901	2.911	2.894	2.904	2.897	2.900	2.904	2.886
([C5MPyrr]+[Tf2N]-)	43	2.838	2.846	2.841	2.840	2.842	2.846	2.839	2.848	2.846	2.844	2.889
([C8MPyrr]+[Tf2N]-)	44	2.865	2.874	2.869	2.867	2.872	2.874	2.867	2.874	2.873	2.871	2.890
([TDC]+[Tf2N]-)	45	2.946	2.952	2.951	2.947	2.964	2.943	2.951	2.942	2.950	2.951	2.896
([ChxMPyrr]+[Tf2N]-)	46	2.867	2.878	2.870	2.869	2.873	2.875	2.869	2.876	2.873	2.873	2.902
([BMPip]+[(Tf)2N]-)	47	2.844	2.852	2.848	2.846	2.849	2.853	2.845	2.854	2.853	2.850	2.903
([MBIm]+[Tf2N]-)	48	2.868	2.874	2.871	2.868	2.875	2.860	2.871	2.864	2.867	2.871	2.907
([ChxPyr]+[Tf2N]-)	49	2.925	2.934	2.928	2.926	2.938	2.918	2.929	2.920	2.924	2.929	2.920

([D2MIm]+[(Tf)2N]-)	50	2.965	2.975	2.969	2.967	2.983	2.965	2.970	2.963	2.968	2.971	2.930
([PeMPip]+[(Tf)2N]-)	51	2.854	2.862	2.858	2.856	2.860	2.863	2.855	2.864	2.863	2.860	2.931
([OiQu]+[(Tf)2N]-)	52	2.965	2.973	2.968	2.966	2.983	2.954	2.971	2.955	2.962	2.968	2.934
([HMPip]+[(Tf)2N]-)	53	2.862	2.871	2.866	2.864	2.869	2.871	2.864	2.872	2.871	2.868	2.935
([MOIm]+[Tf2N]-)	54	2.917	2.925	2.920	2.918	2.930	2.913	2.921	2.914	2.918	2.921	2.938
([DoMIm]+[(Tf)2N]-)	55	2.941	2.950	2.945	2.943	2.957	2.939	2.946	2.939	2.943	2.946	2.943
([OE3Am]+[(Tf)2N]-)	56	2.928	2.937	2.933	2.931	2.944	2.935	2.932	2.933	2.937	2.935	2.944
([Quin6]+[(Tf)2N]-)	57	2.894	2.905	2.897	2.896	2.904	2.901	2.896	2.901	2.900	2.900	2.960
([4-MBPy]+[Tf2N]-)	58	2.880	2.886	2.883	2.880	2.888	2.871	2.883	2.875	2.878	2.883	2.975
([Quin8]+[(Tf)2N]-)	59	2.902	2.913	2.906	2.905	2.913	2.910	2.905	2.909	2.909	2.909	2.979
([MO3Am]+[(Tf)2N]-)	60	2.906	2.915	2.911	2.909	2.919	2.914	2.909	2.913	2.915	2.913	3.001

Figure S1. Descriptor VE2_A (average coefficient of the last eigenvector from the distance matrix) values for the cyclohexane data series cations.

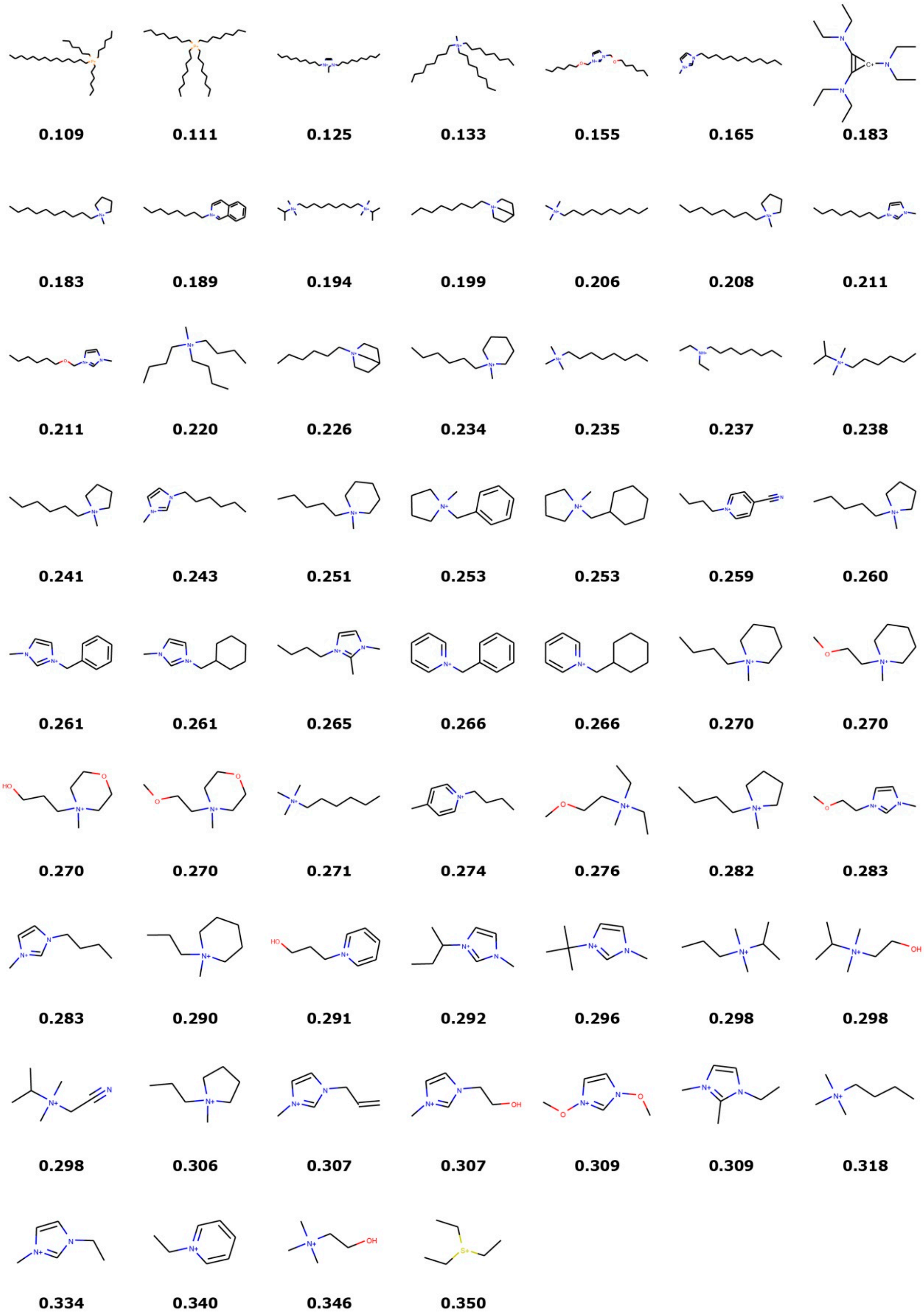

Figure S2. Descriptor ATSC0s (centered Moreau-Broto autocorrelation of lag 0 weighted by intrinsic state) values for the cyclohexane data series cations.

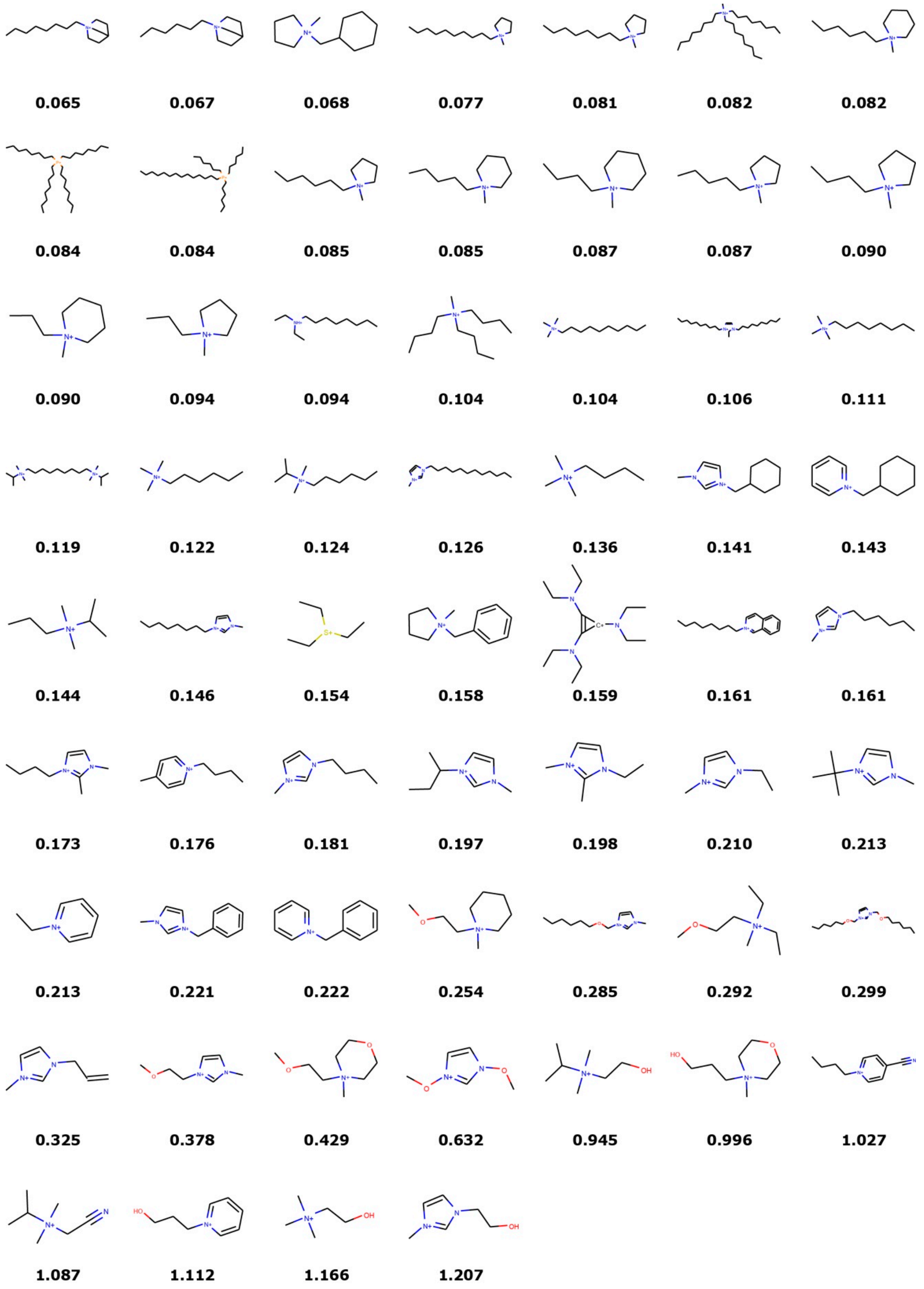

Figure S3. Descriptor Xpc-4d (4-ordered Chi path-cluster weighted by sigma electrons) values for the cyclohexane data series cations.

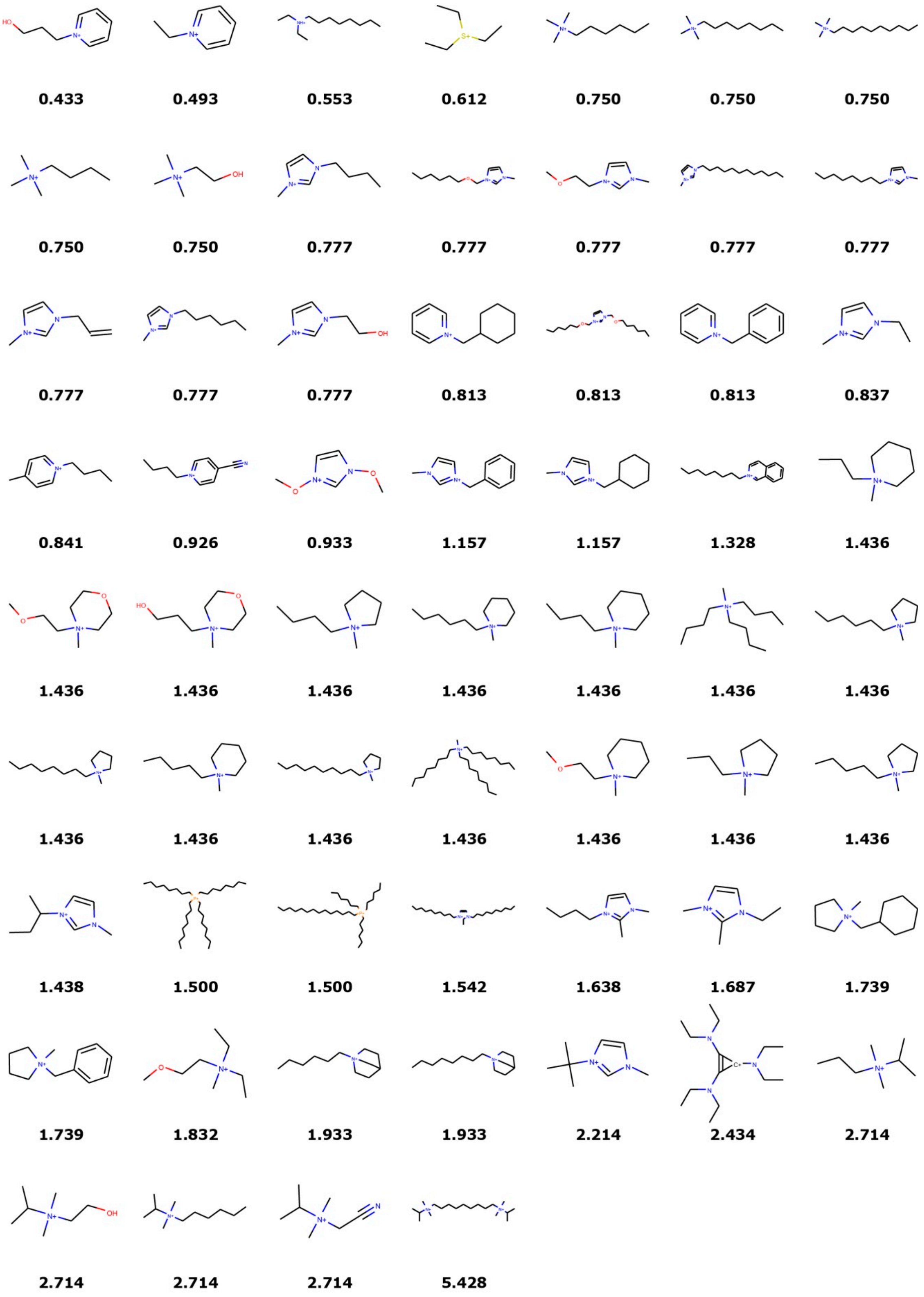

Figure S4. Descriptor AATSC2dv (averaged and centered moreau-broto autocorrelation of lag 2 weighted by valence electrons) values for the hexane data series cations.

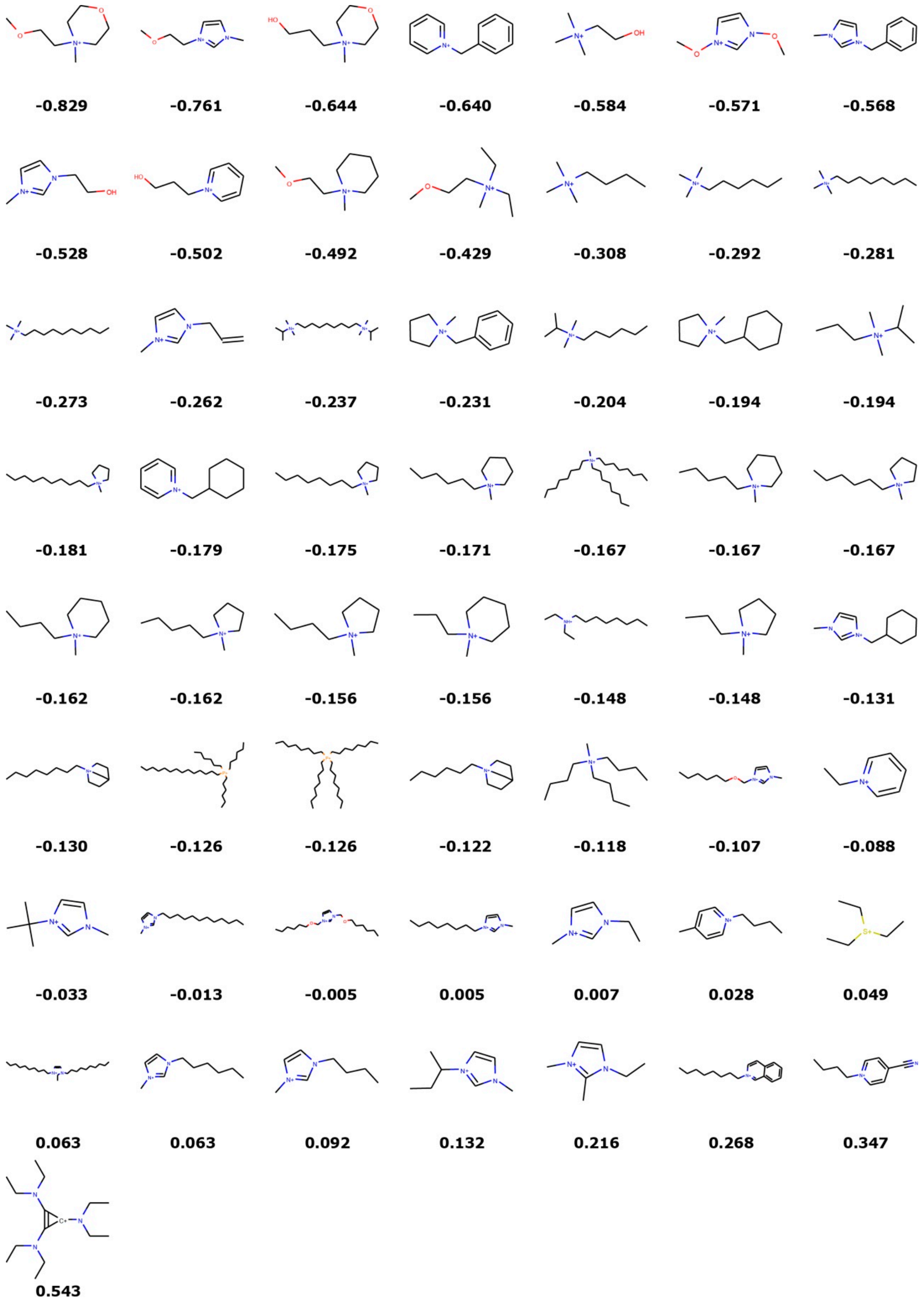

Figure S5. Descriptor AATS7s (averaged moreau-broto autocorrelation of lag 7 weighted by intrinsic state) values for the hexane data series cations.

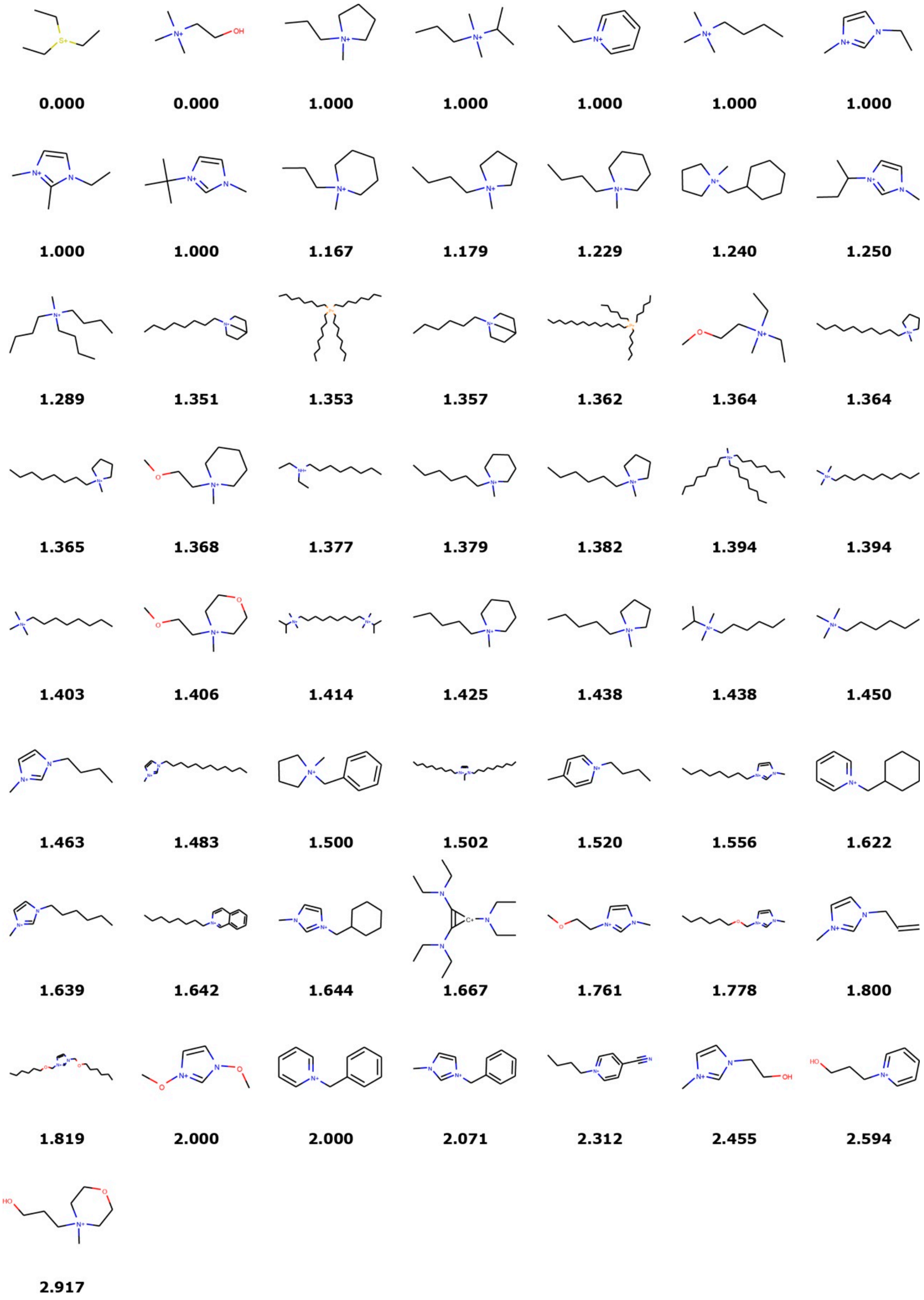

Figure S6. Descriptor GATS7Z (geary coefficient of lag 7 weighted by atomic number) values for the cyclohexane data series cations.

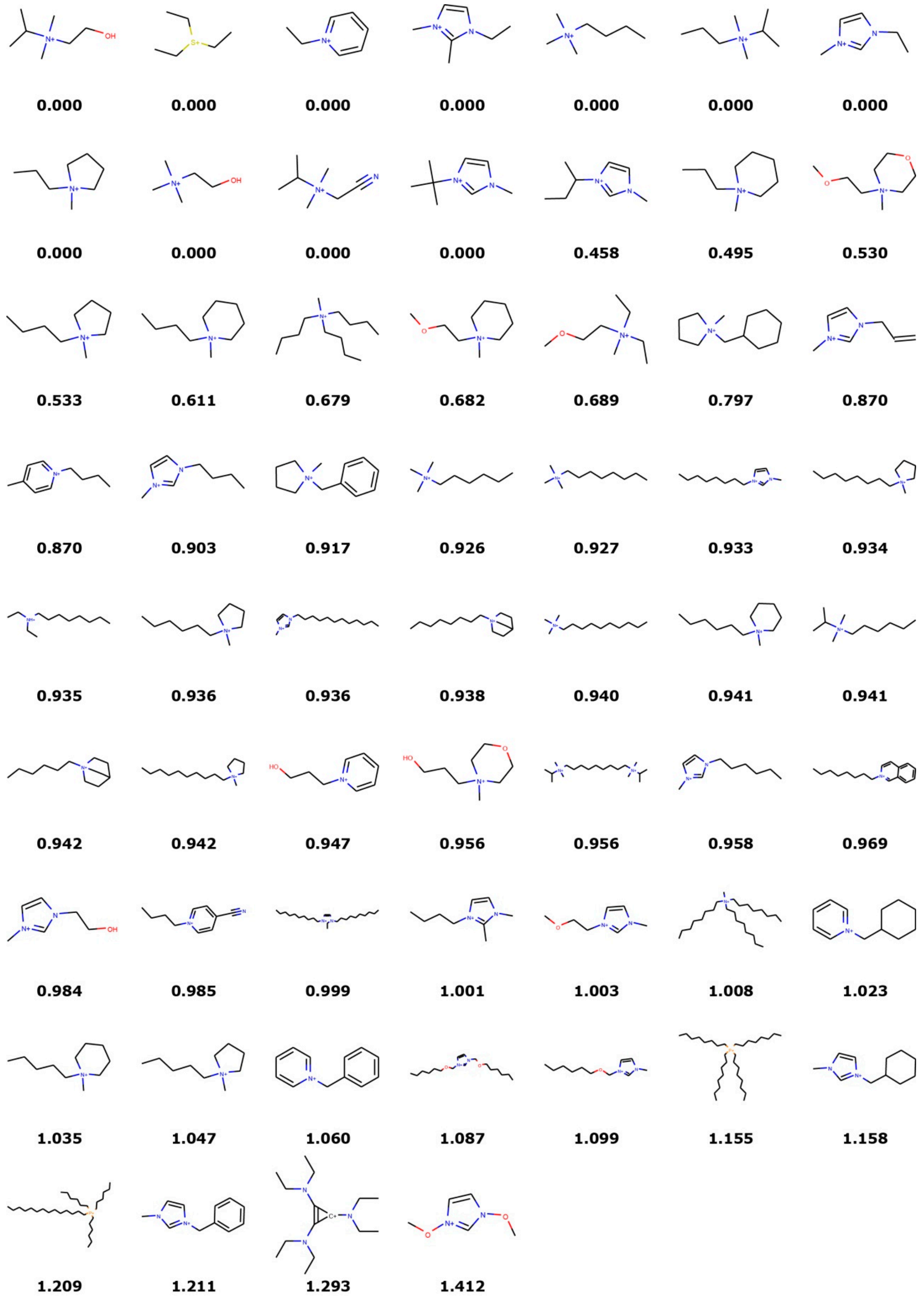

Figure S7. Descriptor AATS0s (averaged Moreau-Broto autocorrelation of lag 0 weighted by intrinsic state) values for the benzene data series cations.

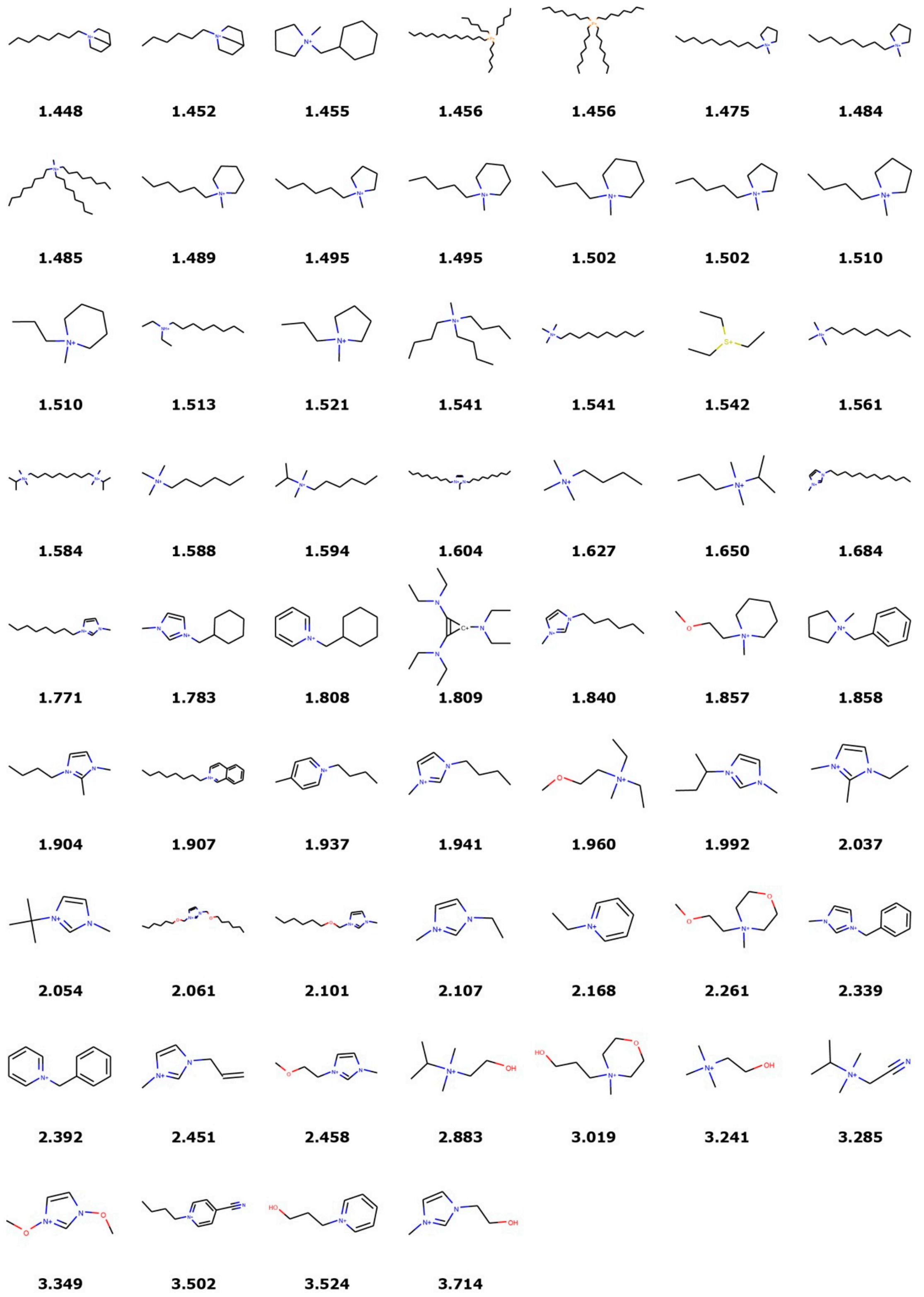

Figure S8. Descriptor GATS2dv (Geary coefficient of lag 2 weighted by valence electrons) values for the benzene data series cations.

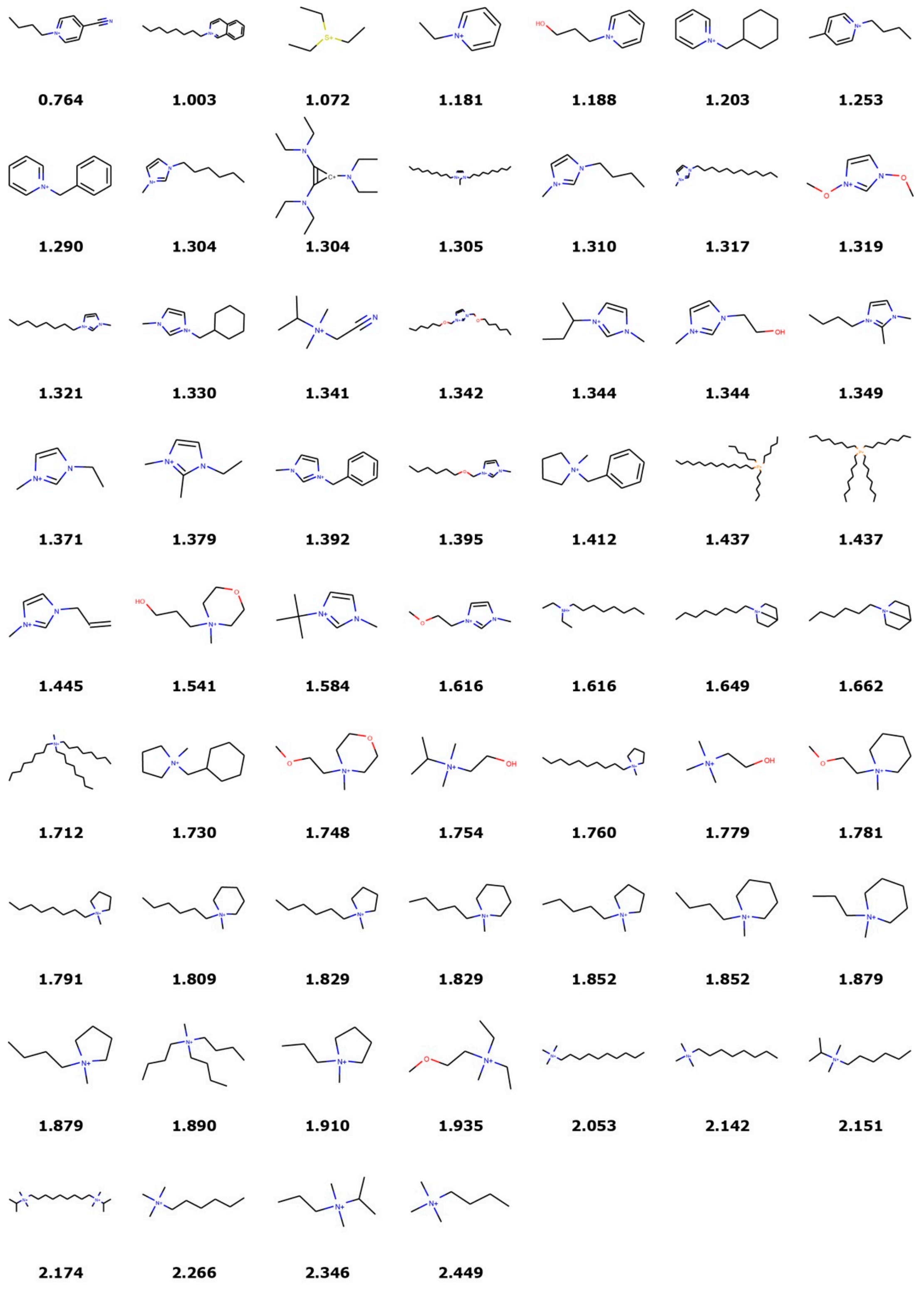

Figure S9. Descriptor GATS3m (Geary coefficient of lag 3 weighted by mass) values for the benzene data series cations.

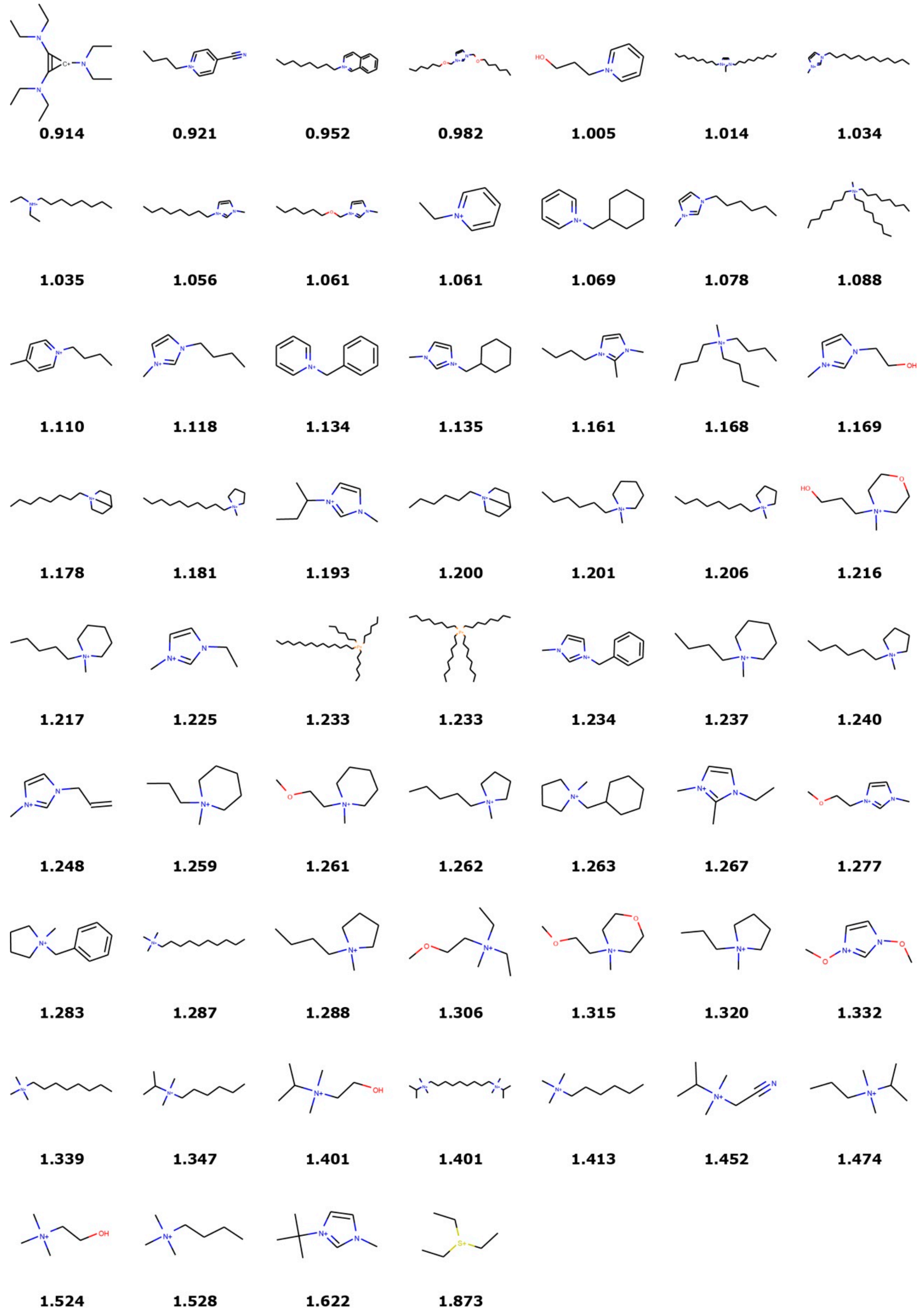

Figure S10. Descriptor SMR_VSA5 (sum of Crippen-Wildman molar refractivity of atoms with van der Waals surface area 2.45 - 2.75) values for the cyclohexane data series cations.

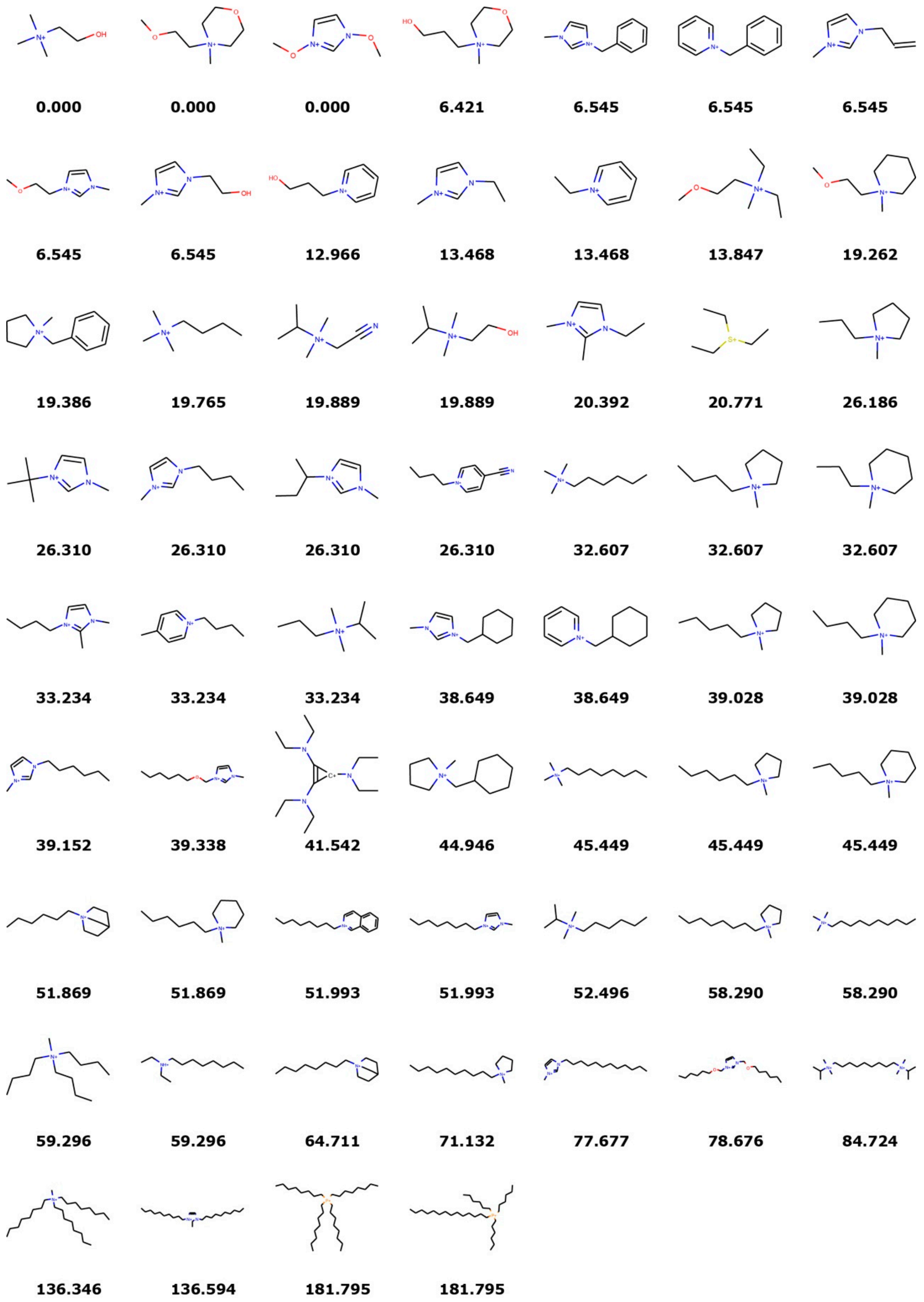

Figure S11. Descriptor AATSC0s (averaged and centered Moreau-Broto autocorrelation of lag 0 weighted by intrinsic state) values for the cyclohexane data series cations.

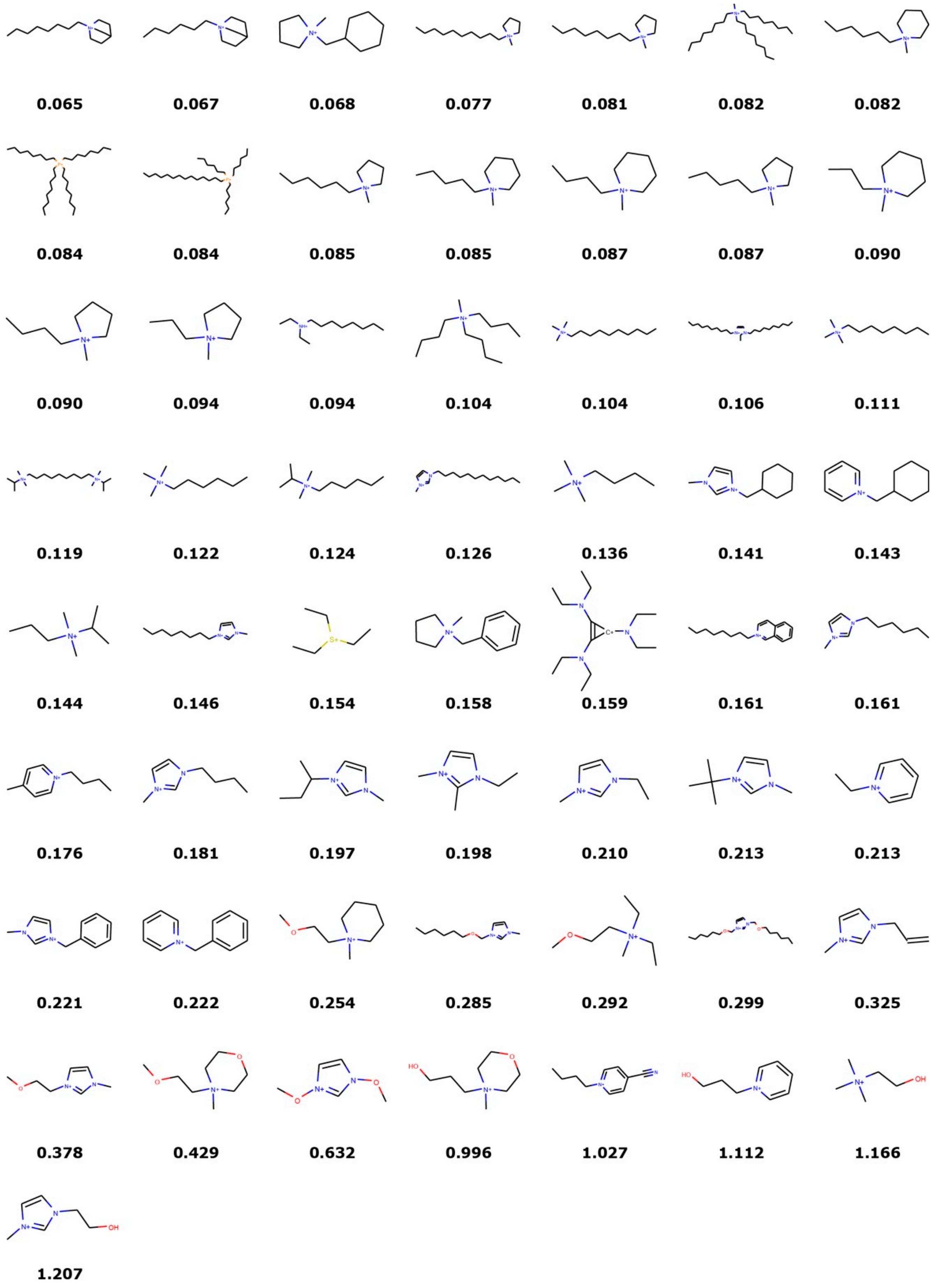

Figure S12. Descriptor Xc-5d (5-ordered Chi cluster weighted by sigma electrons) values for the cyclohexane data series cations.

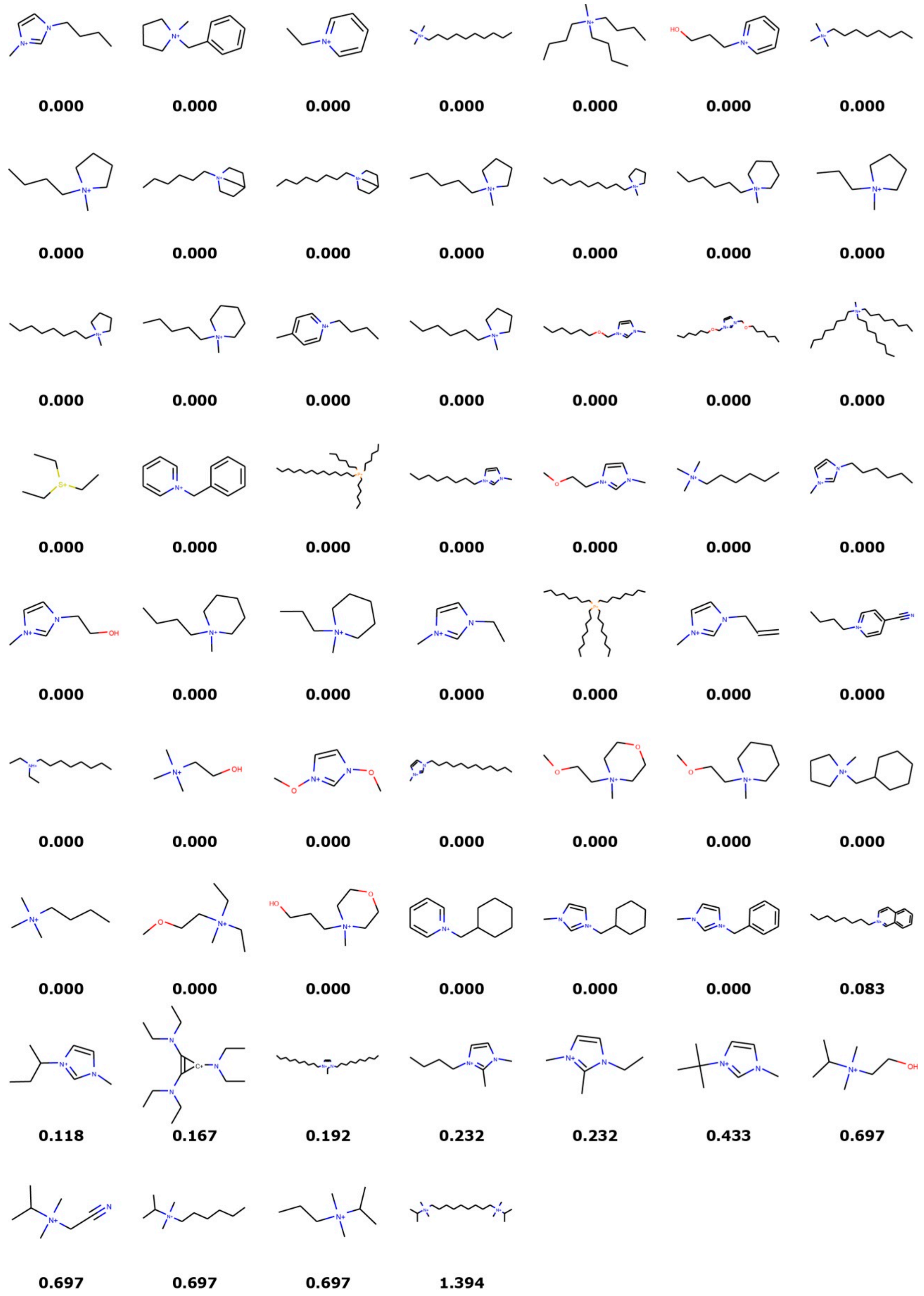

Figure S13. Descriptor AATS6m (averaged Moreau-Broto autocorrelation of lag 6 weighted by mass) values for the hexane data series cations.

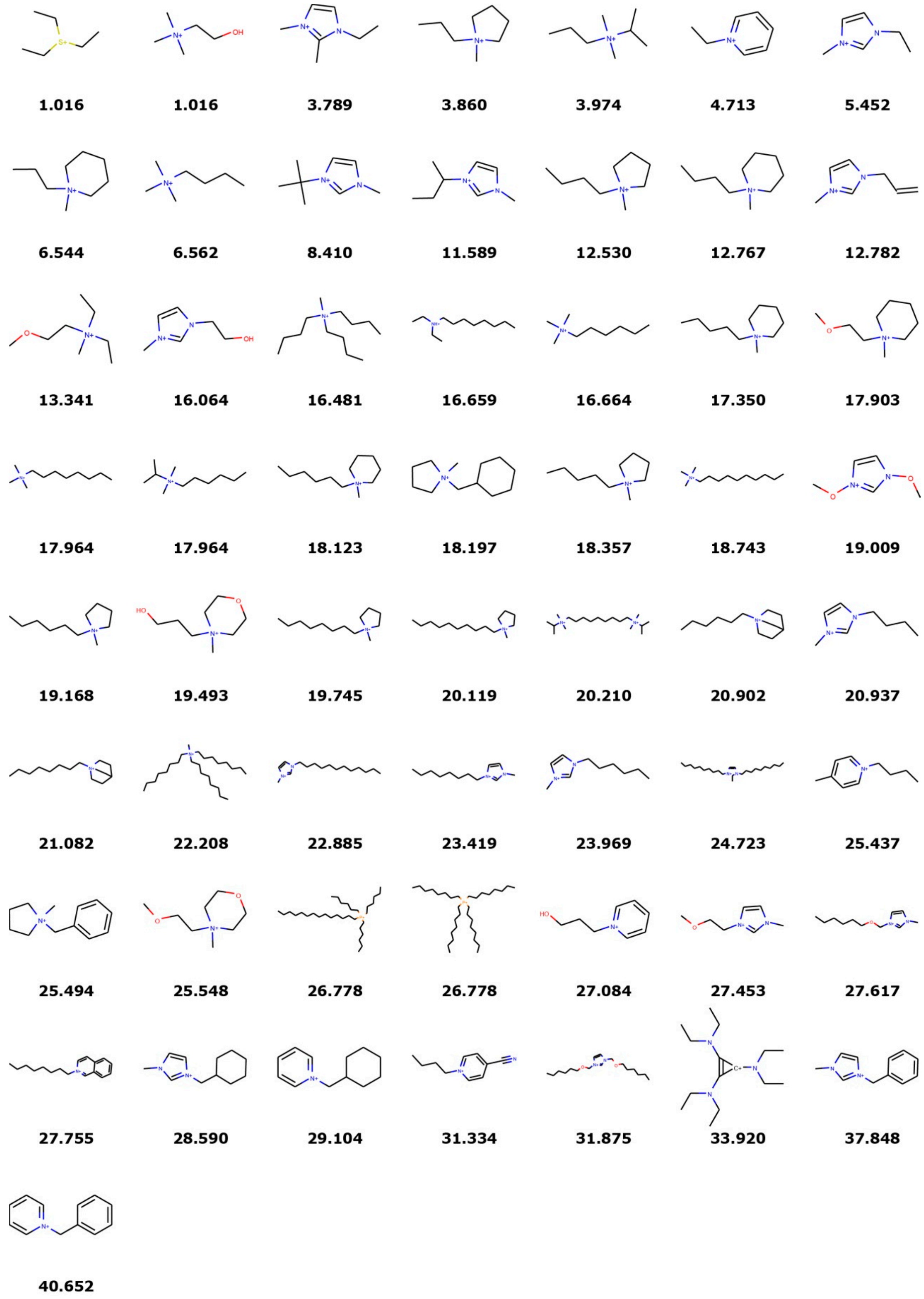

Figure S14. Descriptor AATS7m (averaged Moreau-Broto autocorrelation of lag 7 weighted by mass) values for the cyclohexane data series cations.

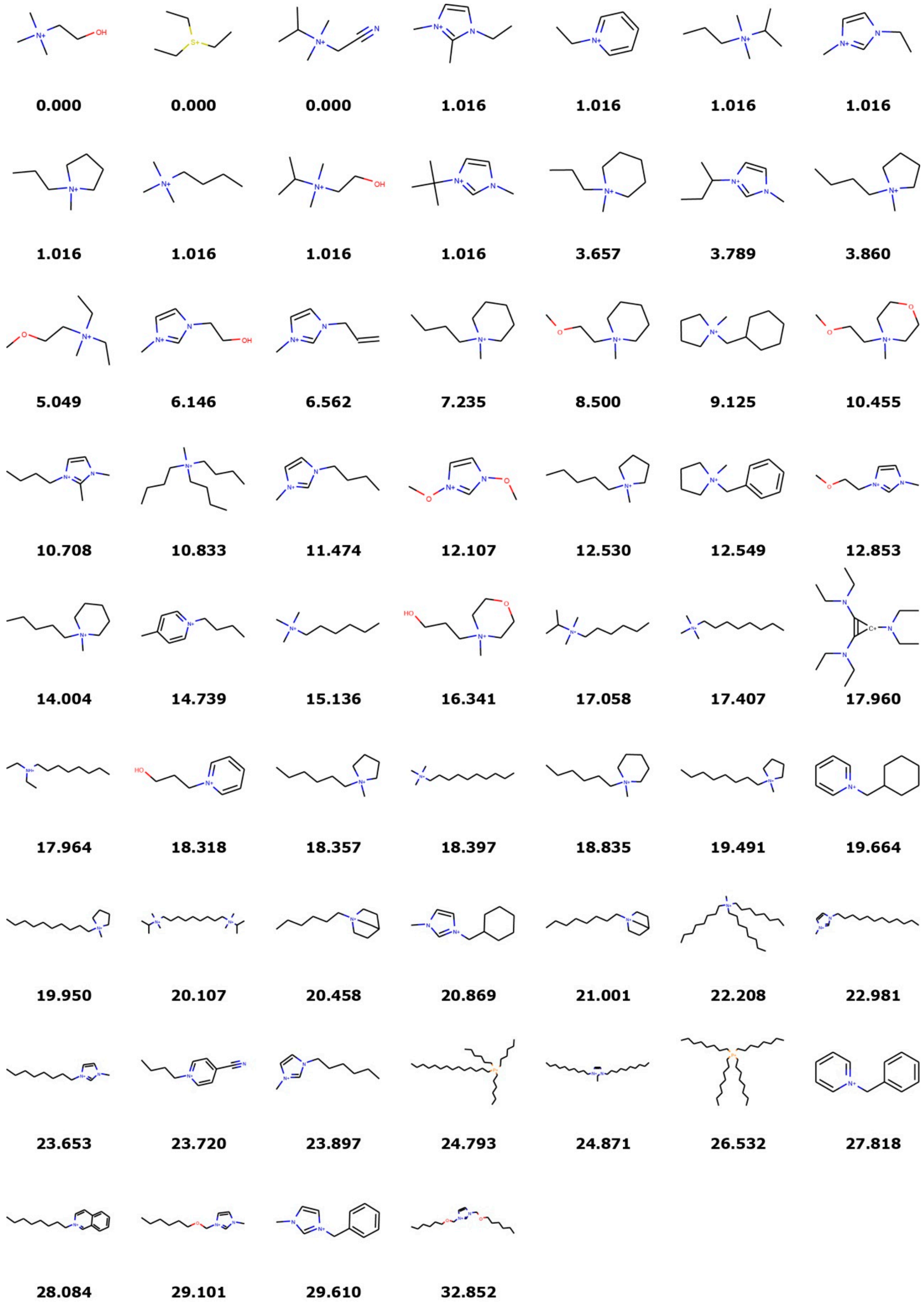

Figure S15. Descriptor SpMAD_D (spectral mean absolute deviation from distance matrix) values for the hexane data series cations.

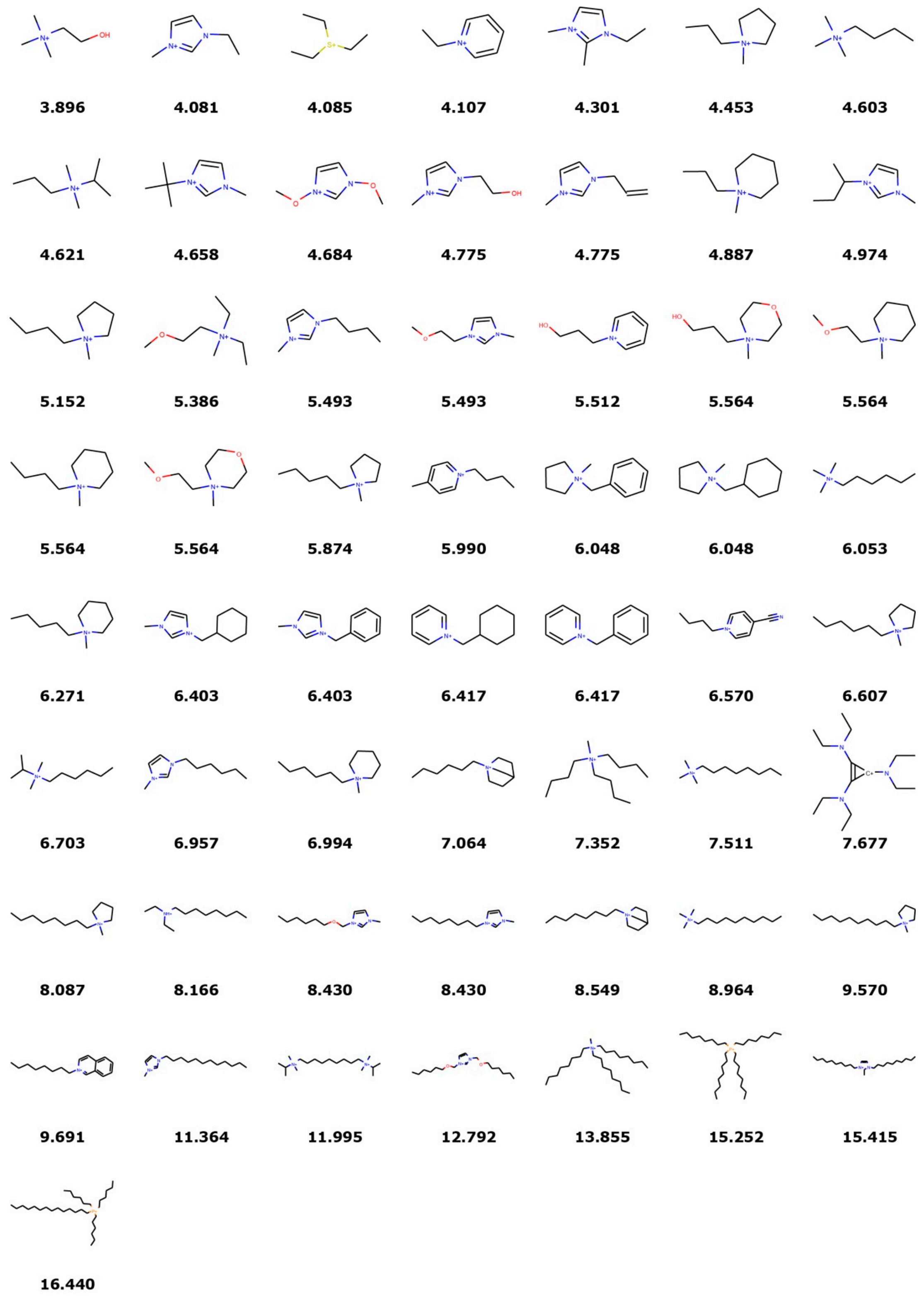

Figure S16. Descriptor Mi (mean of constitutional weighted by ionization potential) values for the benzene data series cations.

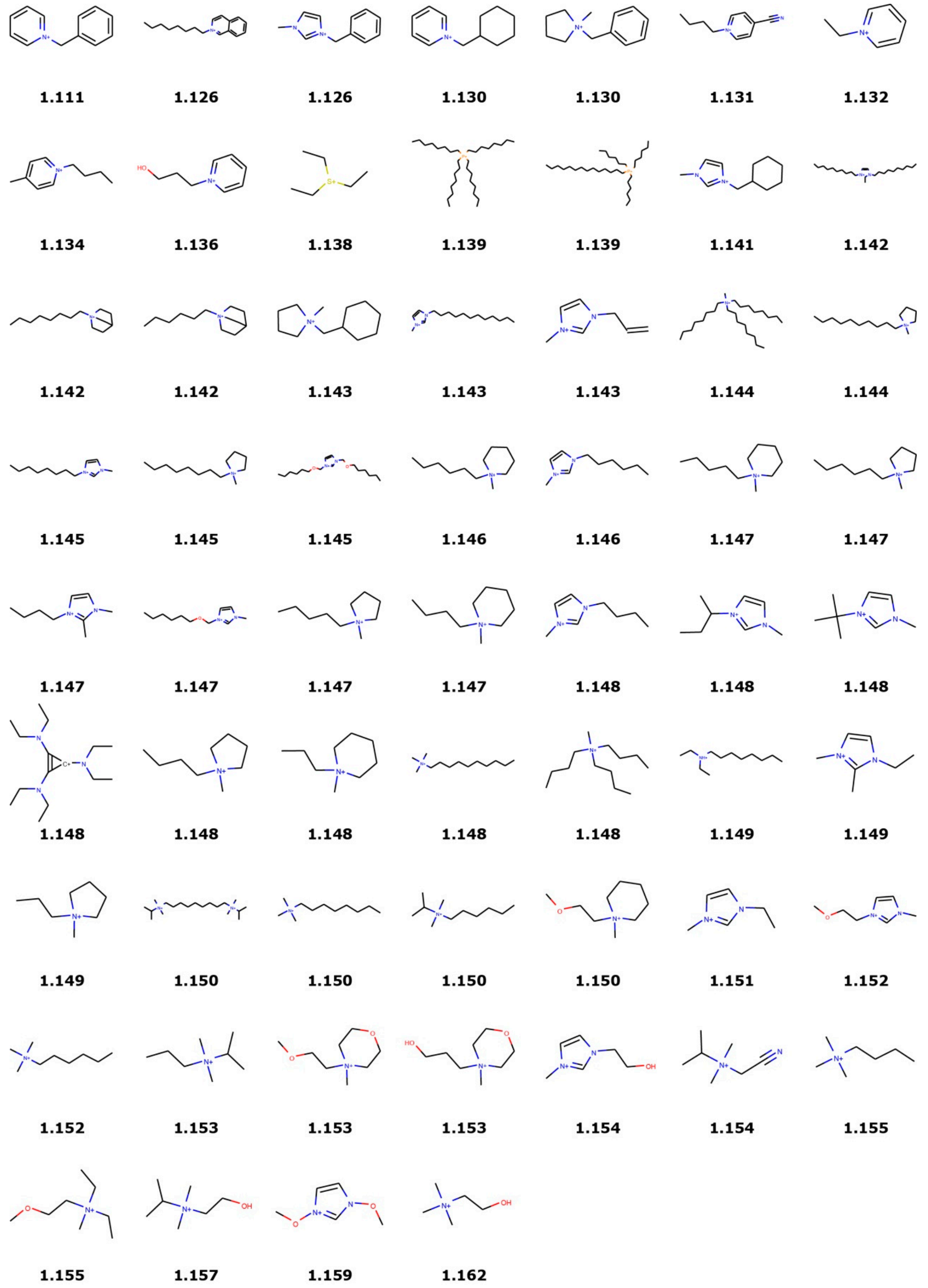

Figure S17. Descriptor ATSC1s (centered Moreau-Broto autocorrelation of lag 1 weighted by intrinsic state) values for the benzene data series cations.

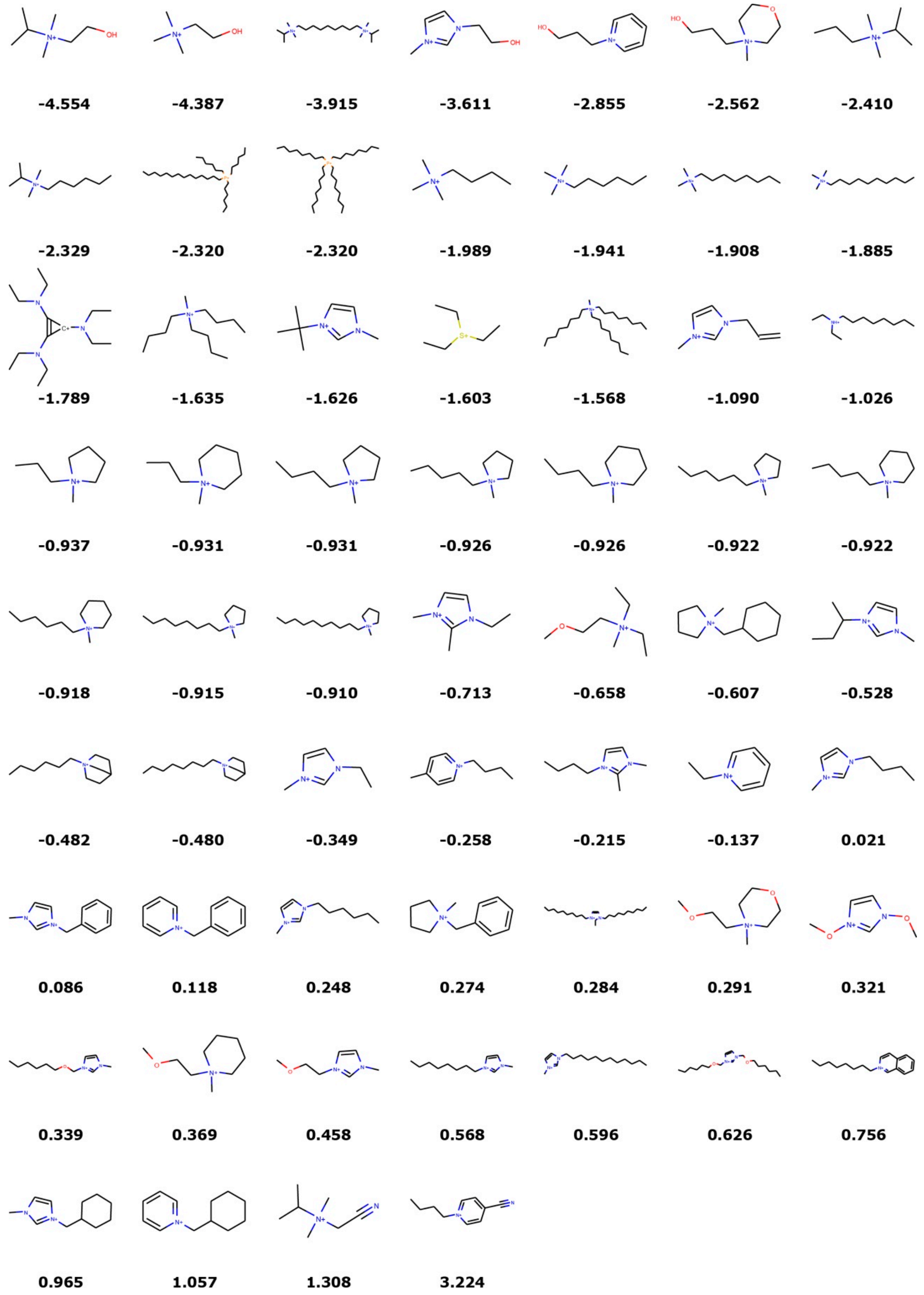

Figure S18. Descriptor GATS2pe (Geary coefficient of lag 2 weighted by Pauling electronegativity) values for the benzene data series cations.

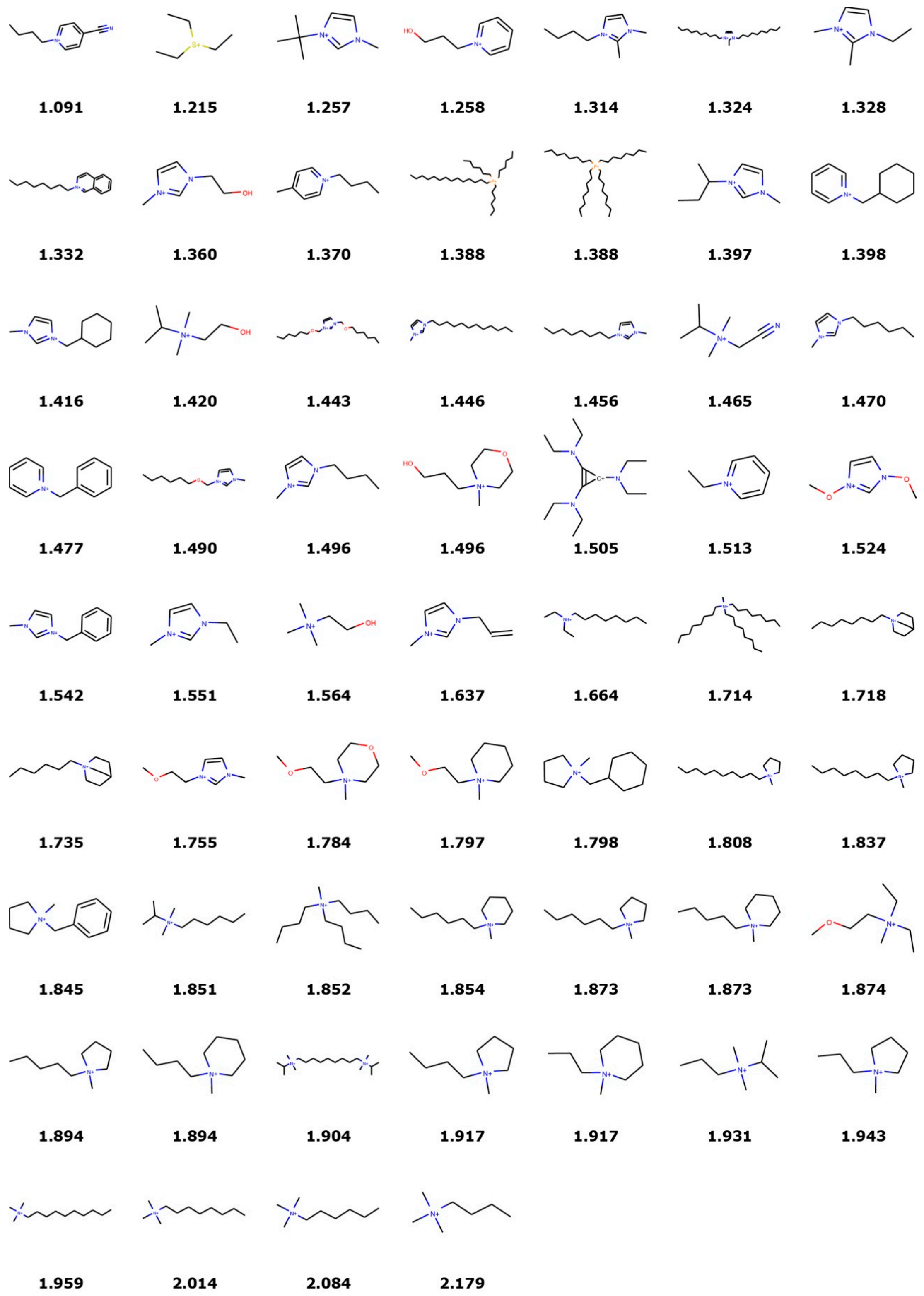

Figure S19. Descriptor AATSC8i (averaged and centered Moreau-Broto autocorrelation of lag 8 weighted by ionization potential) values for the benzene data series cations.

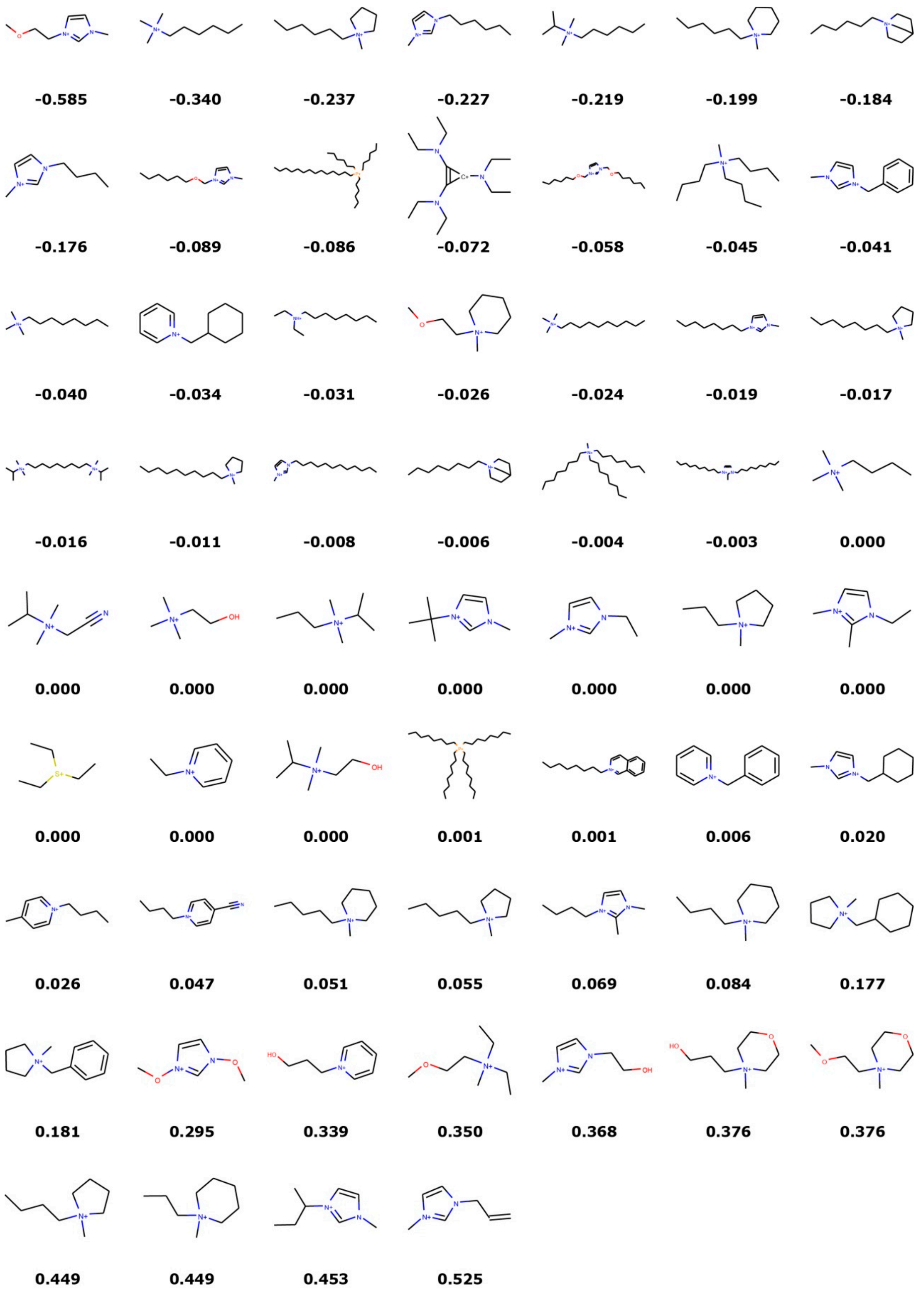

Figure S20. Descriptor GATS1s (Geary coefficient of lag 1 weighted by intrinsic state) values for the hexane data series cations.

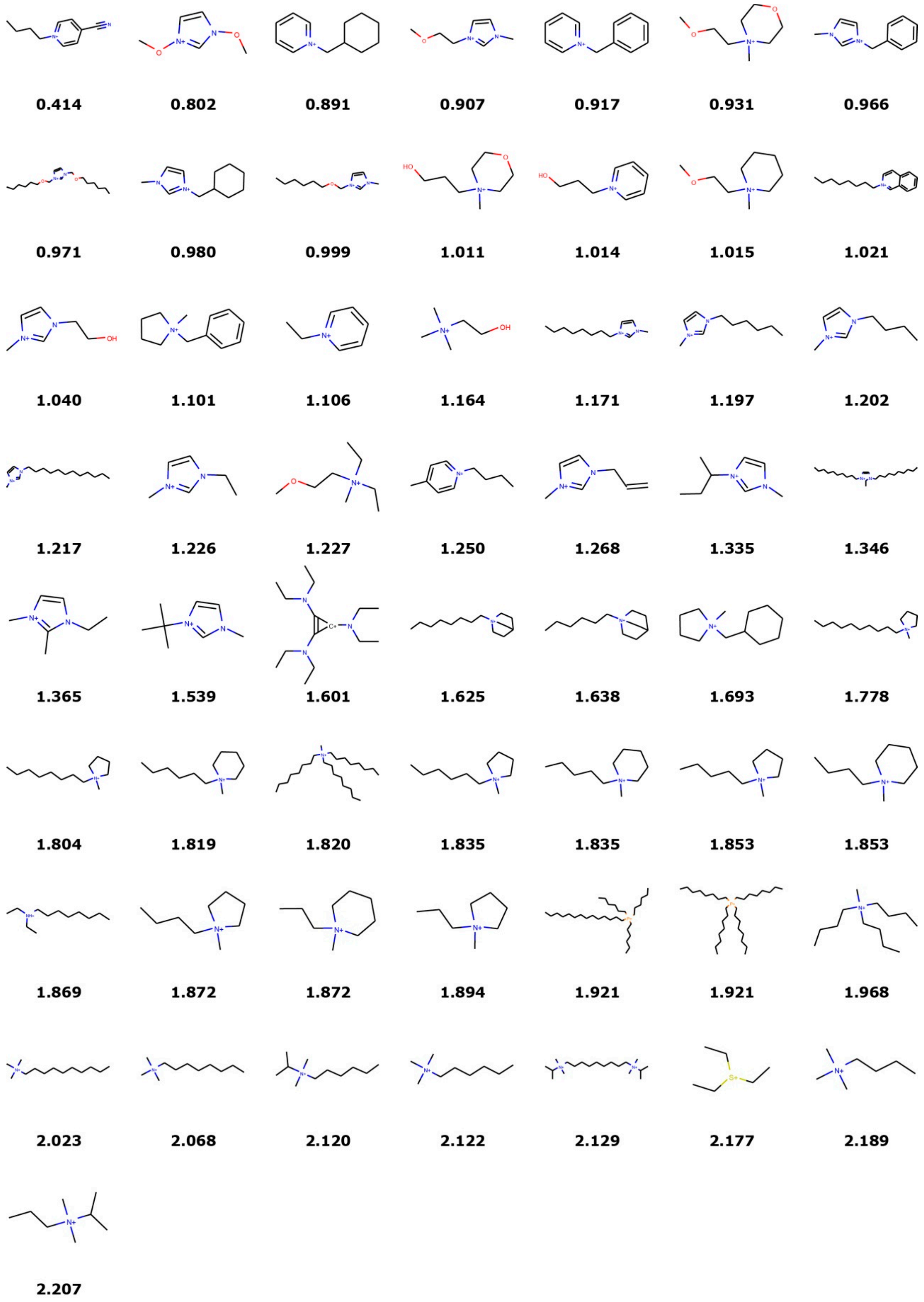

Figure S21. Descriptor ATSC1are (centered Moreau-Broto autocorrelation of lag 1 weighted by Allred-Rochow EN) values for the hexane data series cations.

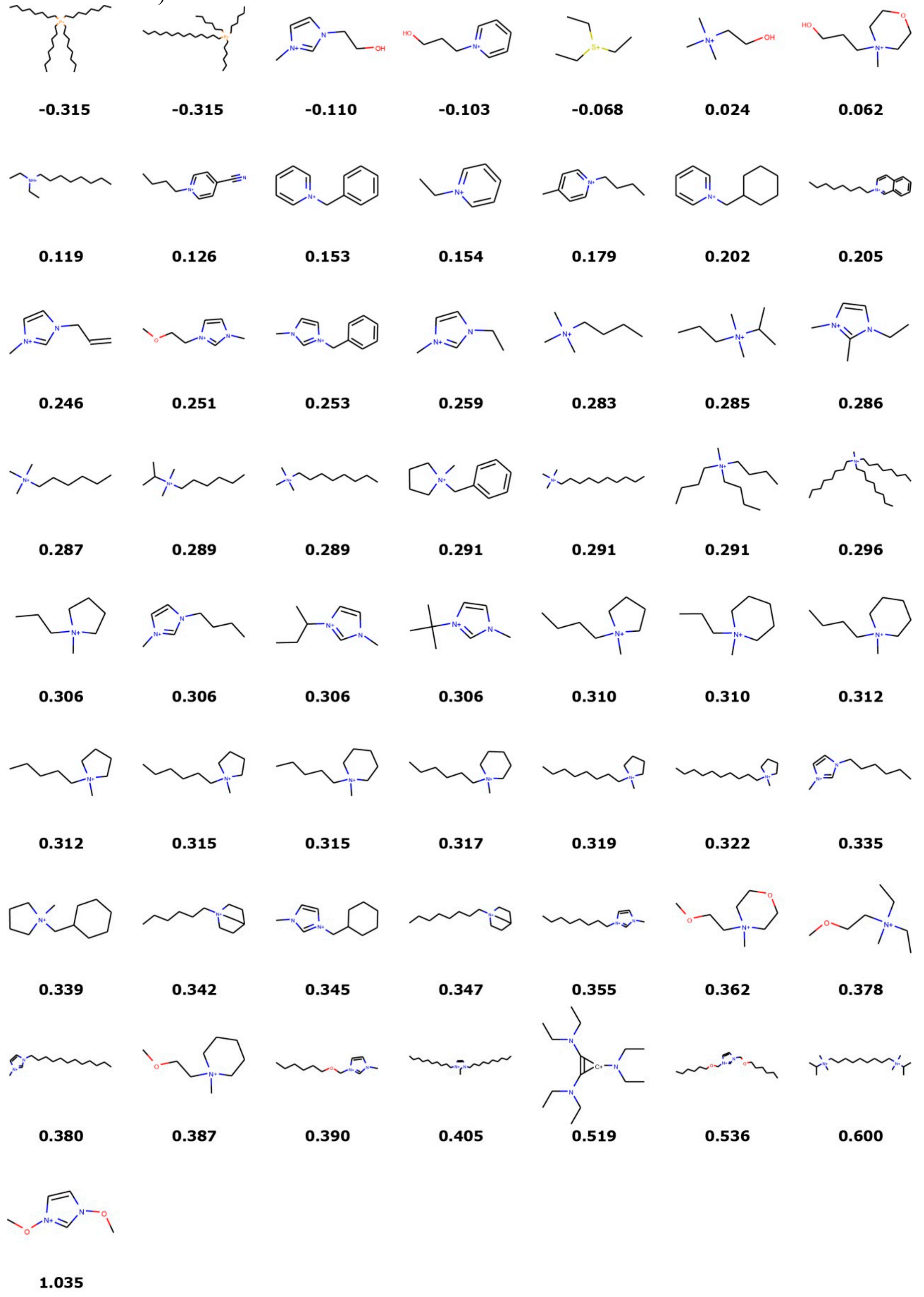

Figure S22. Descriptor SLogP (Wildman-Crippen LogP) values for the cyclohexane data series cations.

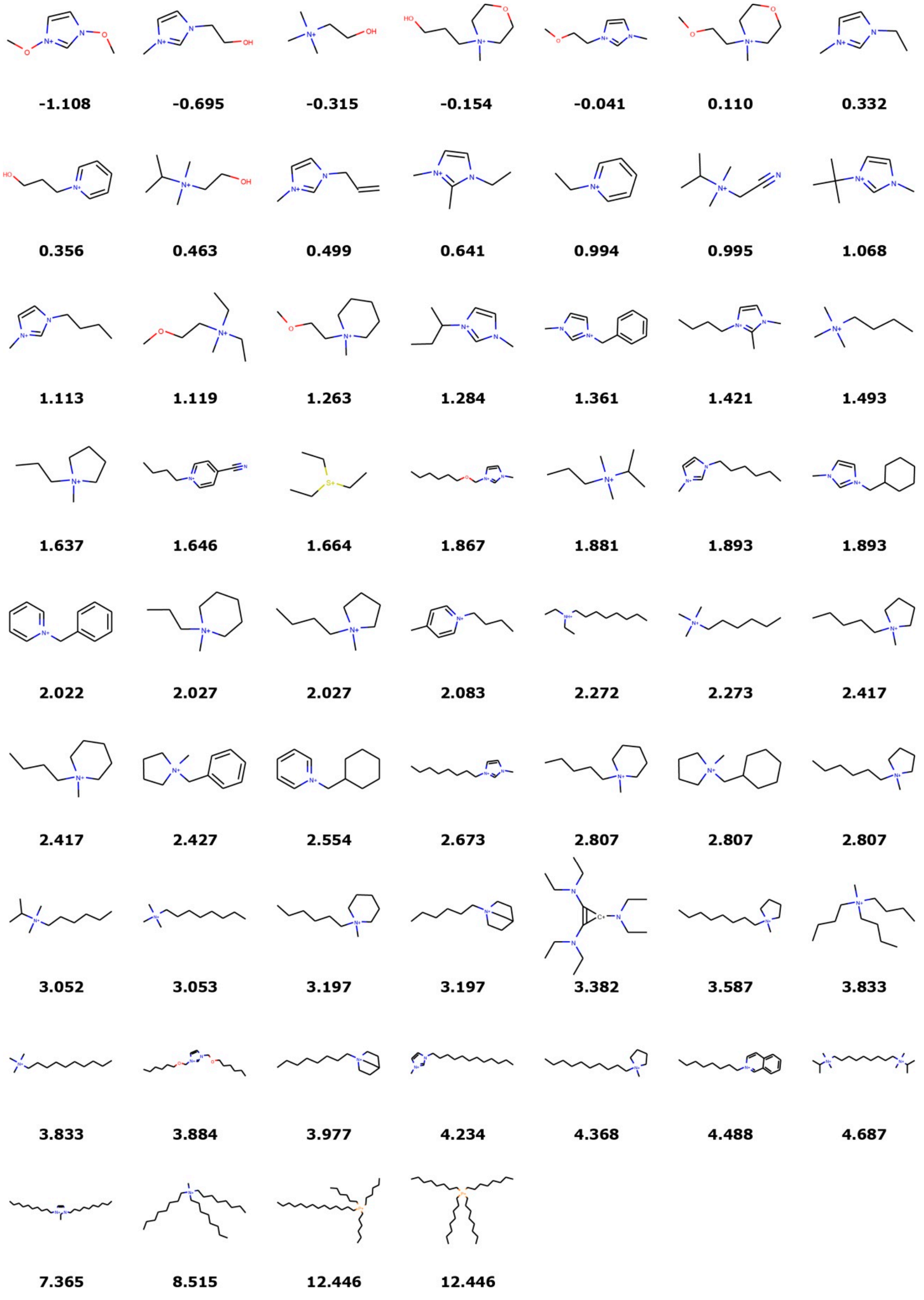

Figure S23. Descriptor Xpc-4dv (4-ordered Chi path-cluster weighted by valence electrons) values for the cyclohexane data series cations.

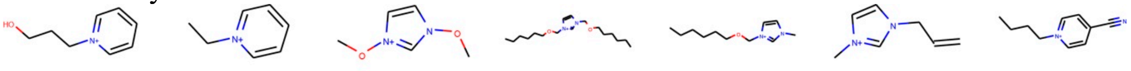

0.219 0.254 0.264 0.325 0.329 0.345 0.353

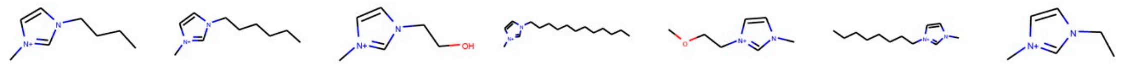

0.359 0.359 0.359 0.359 0.365 0.365 0.390

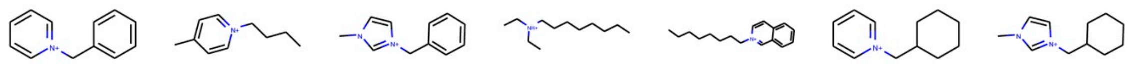

0.390 0.412 0.535 0.553 0.589 0.595 0.740

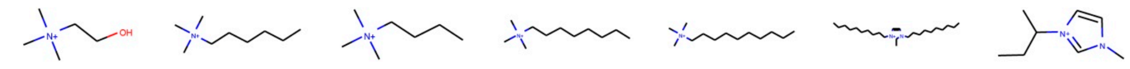

0.750 0.750 0.750 0.750 0.750 0.820 0.863

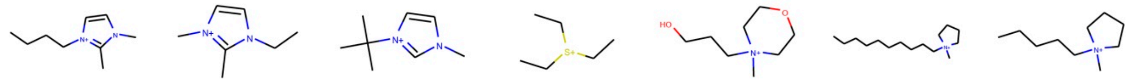

0.867 0.893 1.362 1.423 1.436 1.436 1.436

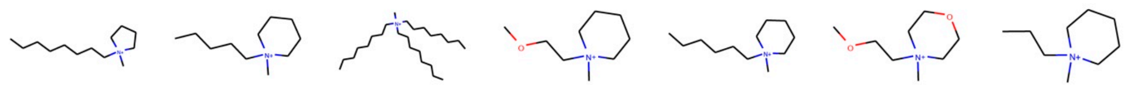

1.436 1.436 1.436 1.436 1.436 1.436 1.436

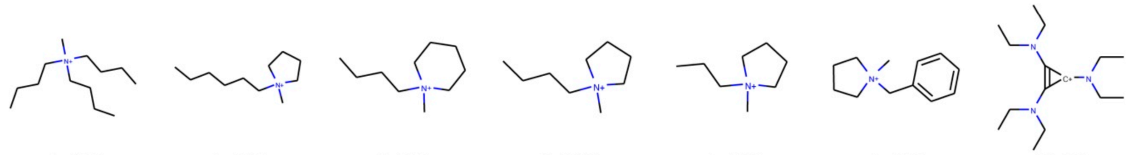

1.436 1.436 1.436 1.436 1.436 1.491 1.522

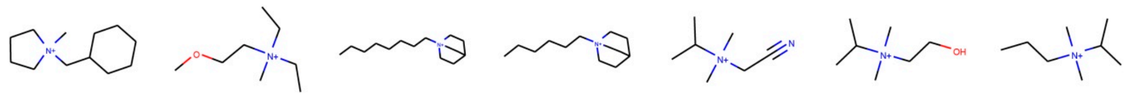

1.739 1.832 1.933 1.933 2.556 2.714 2.714

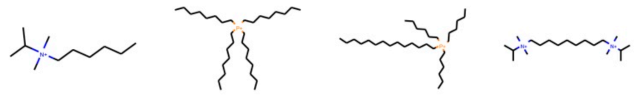

2.714 4.500 4.500 5.428

Figure S24. Descriptor AATSC6se (averaged and centered Moreau-Broto autocorrelation of lag 6 weighted by Sanderson electronegativity) values for the cyclohexane data series cations.

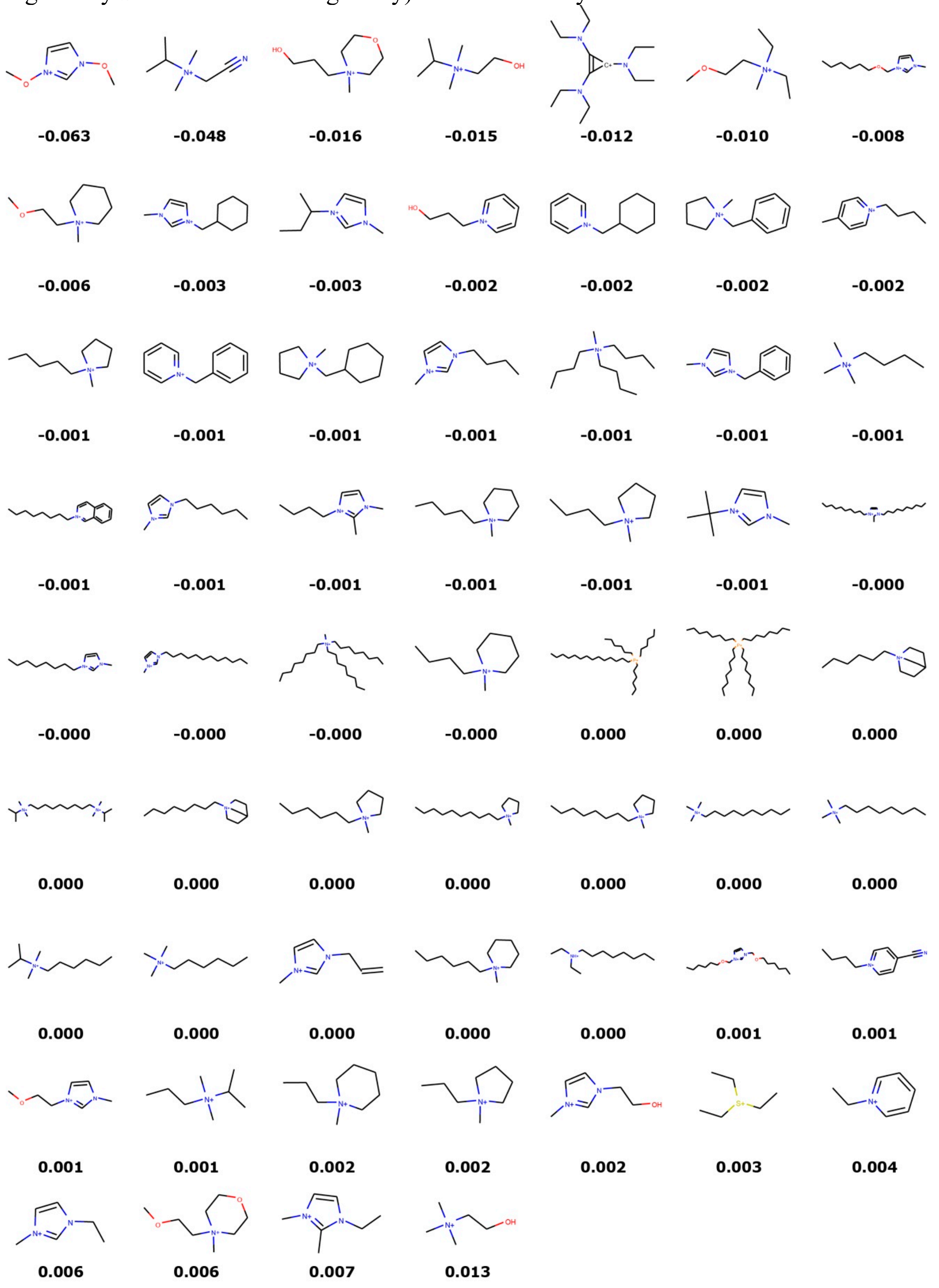

Figure S25. Descriptor MATS8c (Moran coefficient of lag 8 weighted by Gasteiger charge) values for the cyclohexane data series cations.

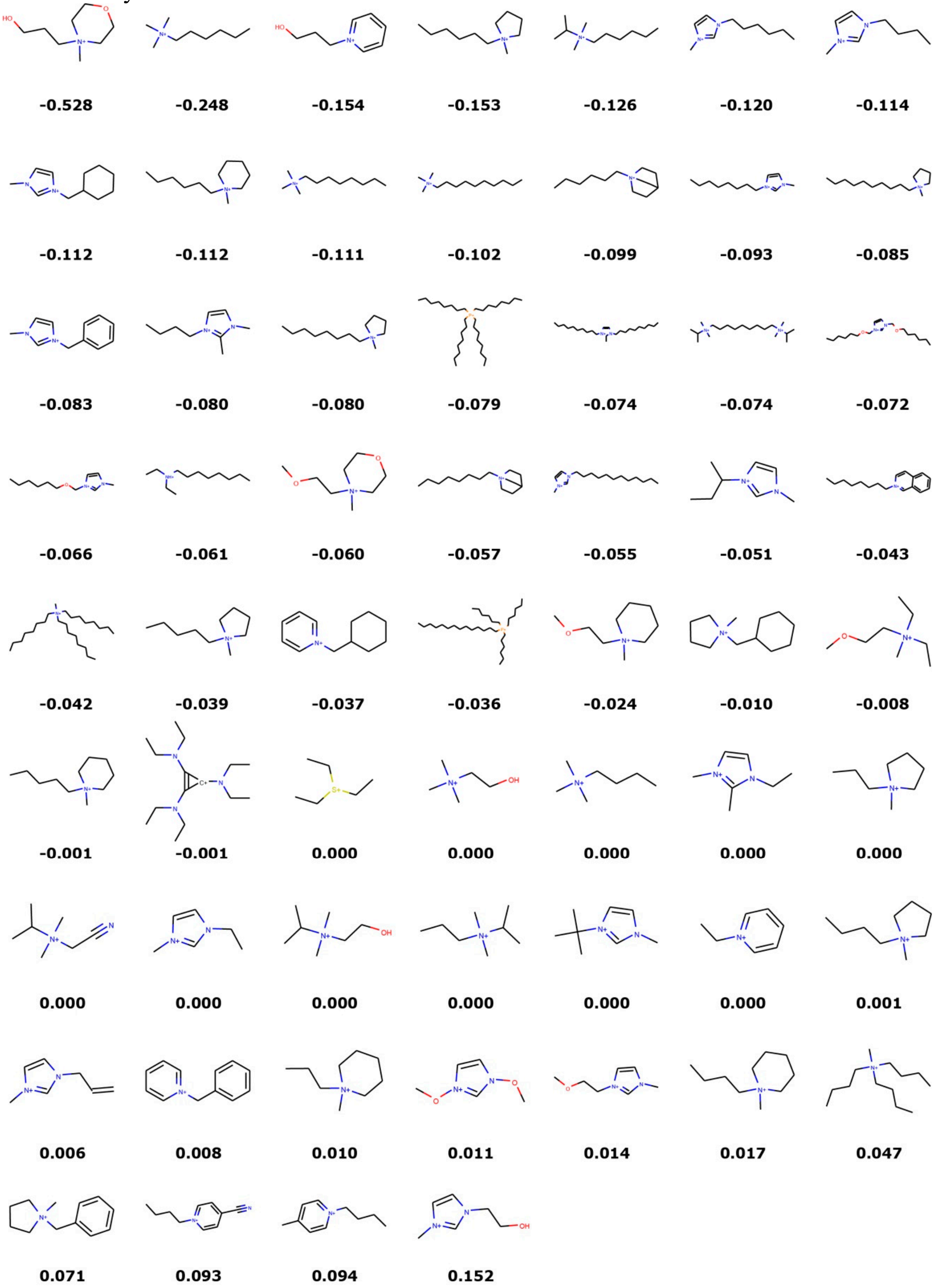

Figure S26. Descriptor GATS3Z (Geary coefficient of lag 3 weighted by atomic number) values for the benzene data series cations.

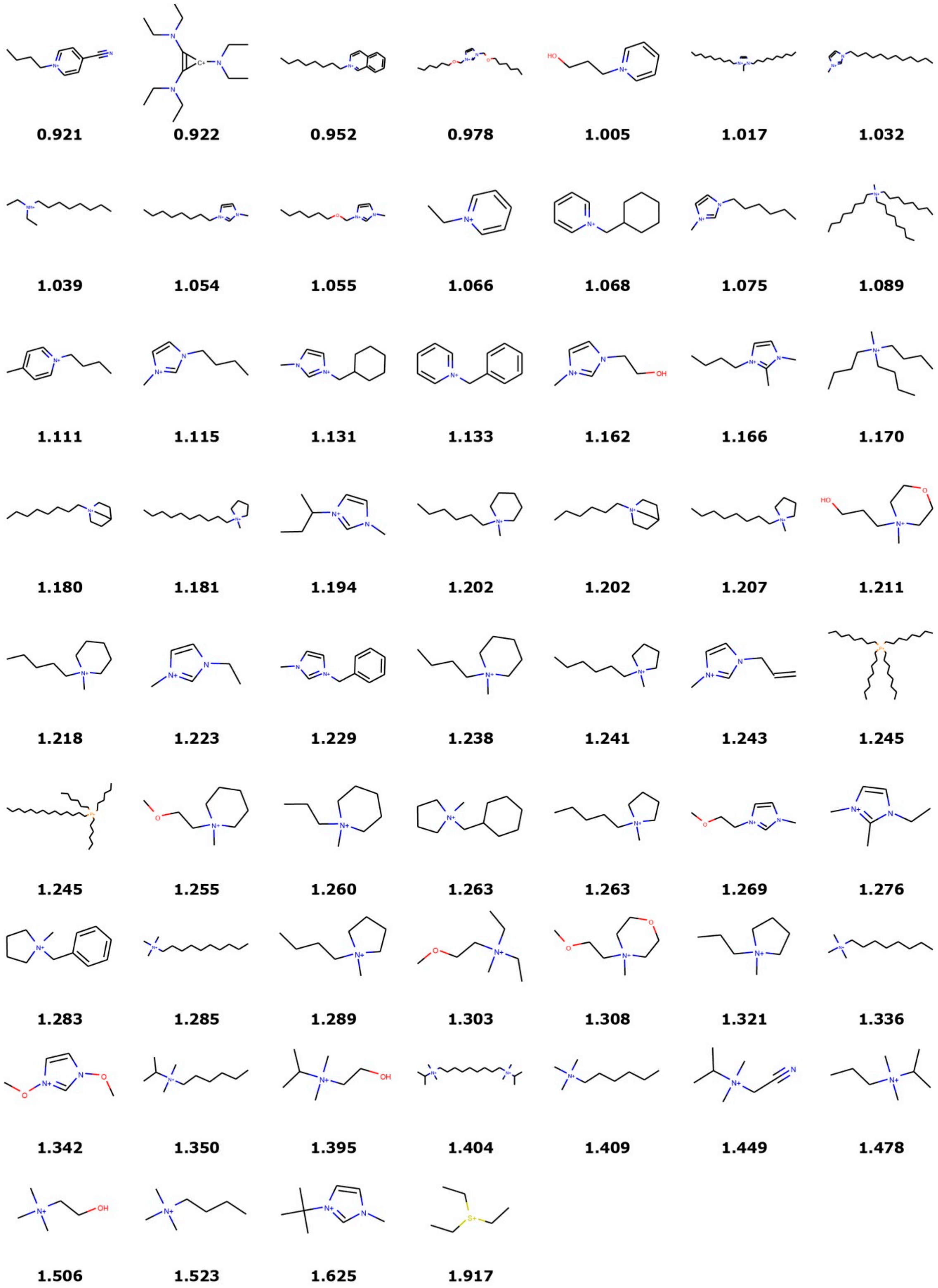

Figure S27. Descriptor GATS3Z (Geary coefficient of lag 3 weighted by atomic number) values for the benzene data series cations.

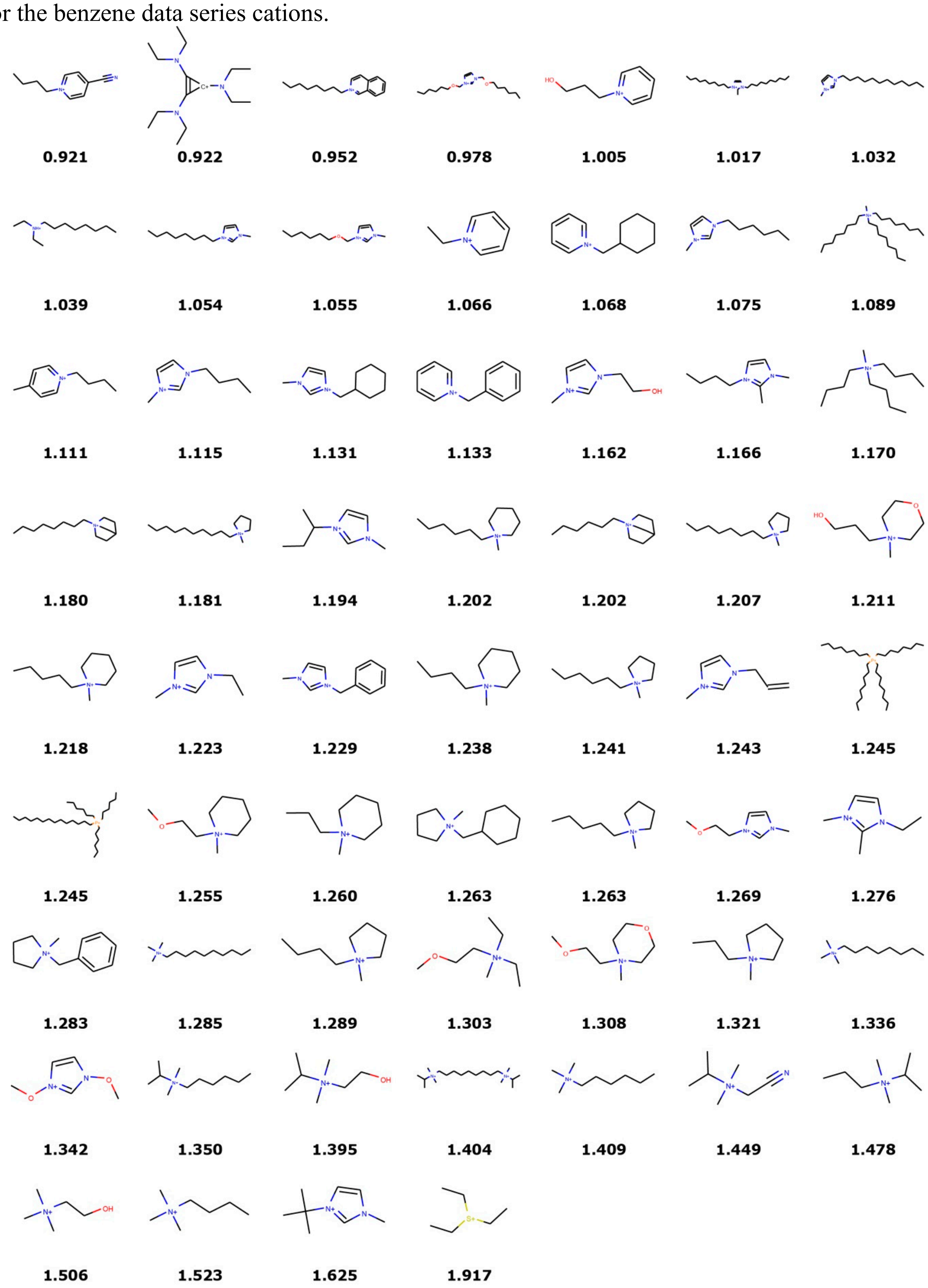

Supplement: Supplementary file 1 [file ijms-23-07534-s001.zip › ijms-1721540-supplementary.pdf]
